# Injectable Hydrogels for Cancer Therapy over the Last Decade

**DOI:** 10.3390/pharmaceutics11090486

**Published:** 2019-09-19

**Authors:** Giuseppe Cirillo, Umile Gianfranco Spizzirri, Manuela Curcio, Fiore Pasquale Nicoletta, Francesca Iemma

**Affiliations:** Department of Pharmacy, Health and Nutritional Sciences, University of Calabria, 87036 Rende (CS), Italymanuela.curcio@unical.it (M.C.); fiore.nicoletta@unical.it (F.P.N.); francesca.iemma@unical.it (F.I.)

**Keywords:** injectable hydrogels, drug delivery, anticancer activity, natural polymers, synthetic polymers, stimuli-responsive materials

## Abstract

The interest in injectable hydrogels for cancer treatment has been significantly growing over the last decade, due to the availability of a wide range of starting polymer structures with tailored features and high chemical versatility. Many research groups are working on the development of highly engineered injectable delivery vehicle systems suitable for combined chemo-and radio-therapy, as well as thermal and photo-thermal ablation, with the aim of finding out effective solutions to overcome the current obstacles of conventional therapeutic protocols. Within this work, we have reviewed and discussed the most recent injectable hydrogel systems, focusing on the structure and properties of the starting polymers, which are mainly classified into natural or synthetic sources. Moreover, mapping the research landscape of the fabrication strategies, the main outcome of each system is discussed in light of possible clinical applications.

## 1. Introduction

**Figure 1:**
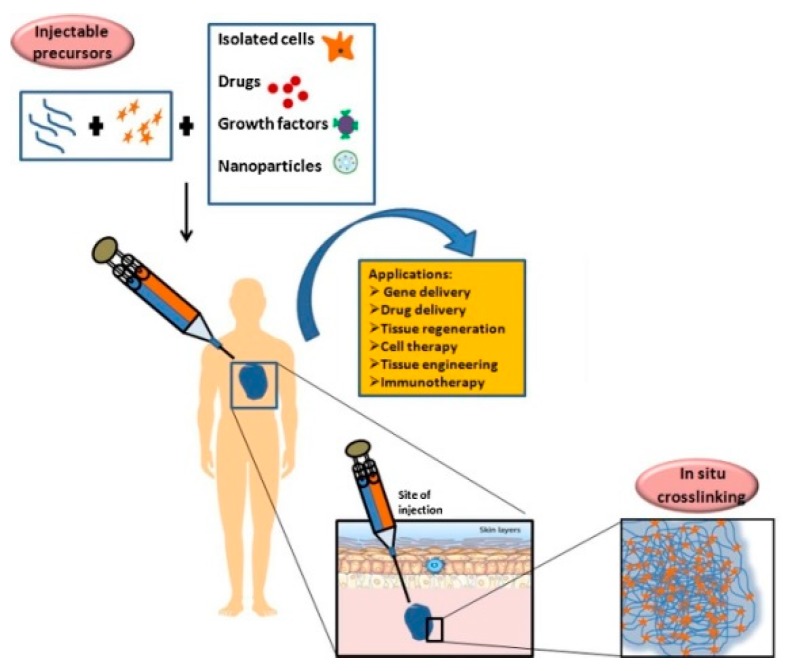
Application of injectable hydrogel systems in biomedical field. Reproduced with permission from [3]. Elsevier, [2018].

Injectable hydrogels can be defined as three-dimensional hydrophilic polymeric networks with a very high affinity for body fluids that may be delivered into body through a catheter or by direct injection with a syringe [1]. Injectable hydrogels have been proposed in the biomedical field as a platform for tissue engineering, as well as for the delivery of therapeutics (Figure 1) [2,3,4].

A gelling mechanism allows injectable hydrogels to be classified into chemically and physically cross-linked hydrogels [5].

Chemical intermolecular cross-linking can be created by the generation of new covalent bonds between polymer chains via photo- or thermo-irradiation [6], or by specific reaction mechanisms involving Schiff’s base formation [7], Diels–Alder cycloaddition [8], Michael-type addition [9], and azide–alkyne (CuAAC) click chemistry [10,11]. The encapsulation of suitable therapeutic agents within the gels during hydrogel formation allows the preparation of three-dimensional structures able to act as a platform for controlled drug delivery or tissue engineering [12]. Chemical hydrogels possess higher mechanical strength (due to high stable crosslink points [13]), longer physical stability, and a prolonged degradation period [14]. Nevertheless, in vivo applications appear reduced due to some potential toxic agents, such as cross-linking monomers, photo-initiators, organic solvents, or catalyzers [2]. Non-covalent bonds such as hydrophobic interactions [15], hydrogen bonding [16], ion cross-linking [17], and host-guest interactions [18] can be exploited in the formation of injectable physical hydrogels. Usually, in the synthesis of this kind of structure, the required mild reaction conditions avoid the generation of any toxic by-products. Furthermore, organic solvents, cross-linking catalysts, or photo-initiation processes are not required during the gelation process [2]. On the contrary, physical hydrogels suffer from some drawbacks compared with the chemically cross-linked formulations, particularly related to bond stability and poor mechanical properties [19].

The mechanical properties of injectable hydrogels are a critical parameter for its function and applications, with the nature of gel being evident by a storage modulus G′ higher than the corresponding values of the loss modulus G″ [20,21]. The resulting mechanical properties of any injectable hydrogels should be adequate to withstand the deformations occurring in the body [22]. The viscosity of the polymer solution is an important parameter that should also be considered in the case of injectable matrices: Precursor aqueous solutions should possess sufficiently low viscosity, or at least adequate shear-thinning properties, to allow for easy injection [23,24,25]. This requirement makes molecular weight control, polymer architecture, as well as chemical composition, very important parameters to be controlled in the design of an effective hydrogel system, which should also allow a homogeneous drug dispersion before the gelation of the cross-linked structure [26]. The U.S. Food and Drug Administration (FDA) fixed the upper limit for any injectable solutions to 0.05 Pa s [27]. Upon gelation, a rapid increase in this value was observed, followed by a leveling off over time [28]. The mechanical properties of the whole hydrogel are strictly dependent on another important structural parameter, such as the porosity (e.g., the space between cross-links). An increased concentration or cross-linking density would enhance the mechanical strength, thus promoting the integrity duration of the hydrogels [29]. Nevertheless, this would determine the hydrogel’s porosity to be significantly reduced, limiting the movement of nutrients and solutions for either the growth of the cell in tissue engineering applications or the modulation of the release profile in drug delivery [30]. Thus, a valuable balance between these parameters should be achieved.

Clinical applications of injectable hydrogels require some fundamental mandatories, such as biodegradability, biocompatibility, stability, non-toxicity, and suitable mechanical and viscoelastic properties. A biocompatible injectable hydrogel should be non-carcinogenic, non-toxic, and should not induce any chronic or adverse physiological response after its degradation. To develop systems with high biocompatibility towards tissues, cells, and body fluids, natural polymers are more suitable than synthetic cross-linked structures due to their subunits, which are more similar to the natural extracellular matrix [31]. Gradual degradation of the hydrogel into biocompatible by-products should also be considered because of their possible accumulation that could generate adverse effects. Usually, carbohydrates, peptides, and nucleic acids naturally degrade in non-toxic by-products [31]. Among the different applications of injectable hydrogels, cancer therapy is one of the most widely explored [32]. The treatment of cancer by systemic chemotherapeutic procedure, indeed, often determines a high level of cytotoxicity [33] and, to overcome this inconvenience, intratumor delivery of therapeutics employing injectable hydrogels can provide a controlled and targeted release within the tumor site [34].

Here, we have reviewed the synthesis and the application of different injectable hydrogels proposed as drug delivery systems for the local delivery of chemotherapeutics. Additionally, stimuli-responsive release of anticancer agents have been treated by the analysis of thermo-, pH-, photo-, or multi-sensitive drug delivery systems, as well as active targeting hydrogels [35]. Based on the main component of the polymer network, herein we have classified the injectable hydrogels reviewed and discussed as synthetic or natural systems. For each class of materials, a summarizing table containing information about composition, carrier and delivery properties, as well as cancer models employed in either in vivo or in vitro experiments has been introduced. Moreover, when available, data about studies in health models have given information about side toxicity and pharmacokinetic profiles. Finally, injectable hydrogels containing nanoparticle systems as functional additive to control the releasing rate have been defined as composite materials, while N/S hybrid hydrogels refer to the simultaneous presence of natural and synthetic polymers within the same polymer structure.

## 2. Synthetic Injectable Hydrogels

### 2.1. Polyphosphazenes

**Figure 2:**
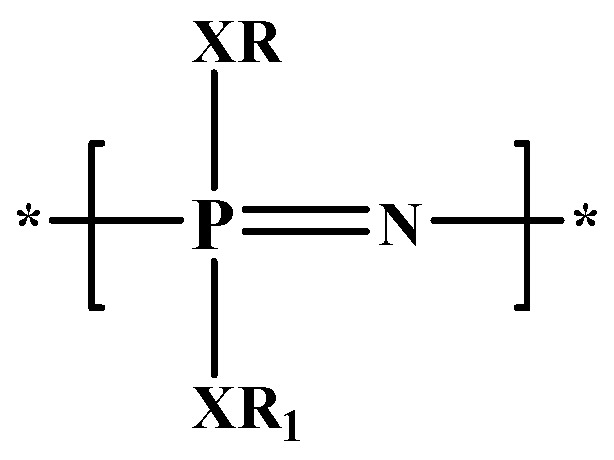
Representation of Polyphosphazenes. X = O, NH; R and R_1_ = Alkyl, Aryl, amino acid.

Polyphosphazenes (PPZs) are a class of hybrid organic–inorganic macromolecules consisting in a linear or branched skeletal structure of repeating phosphorus and nitrogen atoms with alternating single and double bonds [36]. Each phosphorus atom is linked to two organic side groups, ranging from alkyl and aryl moieties to amino acids (Figure 2) [37].

PPZs are obtained via different synthetic routes, with most of the biologically-relevant materials being prepared by a ring-opening polymerization, followed by macromolecular substitution reactions [38]. Either the modification of organic side groups and their ratios, or the attachment of multiple different side groups to the same backbone, allow the preparation of a wide range of PPZs, with finely tuned physical and mechanical properties [39]. The interest in PPZs as materials for the formulation of injectable hydrogels is related to the ability of their aqueous solutions to undergo reversible sol–gel transitions depending on the temperature. In fact, PPZs are in the sol state at room temperature (or below), but they gelate at body temperature. Such transition is tunable by adjusting the balance of hydrophobic to hydrophilic substituents [40]. Furthermore, a growing number of hydrolytically-sensitive PPZ hydrogels have been designed, with negligible toxicity arising from the degradation of by-products generally consisting of H_3_PO_4_, ammonium, and free organic side groups [41]. On the contrary, the employment of cyclic PPZ architecture should be accurately investigated, because such derivatives are characterized by a relatively long time of degradation which can reduce the biomedical applicability [42]. Although a large number of PPZ polymers have not found commercial success [43], several research groups have developed different types of PPZ injectable hydrogels (Table 1). PPZ-based hydrogels were successfully tested for the delivery of cytotoxic drugs or sRNA to solid tumors, both in vitro and in vivo [40,44,45,46,47,48,49,50]. They proved the ability of these systems to extend the release profiles overtime [47] with no-toxicity on healthy mice [46,47] and the possibility to confer targeted behavior [50]. A further upgrade of the use of PPZ was proposed in [51], where the injectable hydrogels consisted of a Camptotechin (CPT) prodrug useful for the treatment of lung and colon cancer cell lines. The insertion of metal ferrite superparamagnetic iron oxide nanoparticles within the hydrogel structure was proved to be a suitable strategy for enabling tumor imaging and magnetic hyperthermia ablation [52,53].

### 2.2. Polaxamers

**Figure 3:**
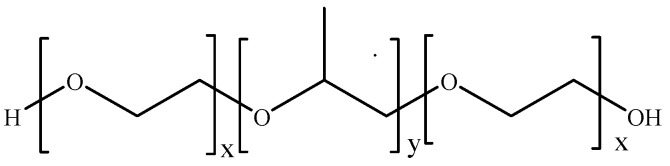
Schematic representation of poloxamers. x: 2–130; y: 15–67.

Poloxamers (also known as Pluronics) are tri-block amphiphilic polymers consisting of poly(ethylene oxide)-poly(propyleneoxide)-poly(ethylene oxide) (PEO-PPO-PEO) repeating units [54]. They are non-ionic surfactants, with physical and chemical properties depending on the molecular weight and hydrophilic (PEO) to hydrophobic (PPO) balance (Figure 3) [55].

Among others, PF127 (PEO/PPO balance 70/30) is one of the most widely employed poloxamers for biomedical applications due to the ability to form either micellar nanocarriers for lipophilic drugs (due to PPO content) or hydrogel networks upon reverse thermal gelation. PF127 water solutions (>20% by weight) show a low-viscosity state at 4 °C, while semisolid gels are obtained upon heating to room or body temperature, probably due to micellar packing and entanglement [56,57].

**Figure 4:**
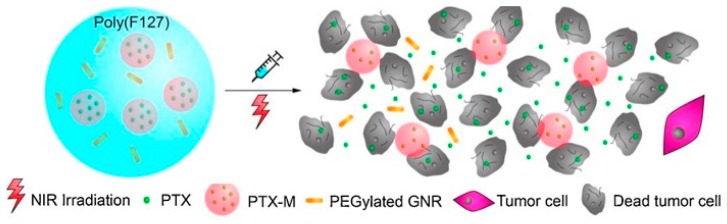
Schematic representation of the PTX-NPs/AuNRs/gel-mediated photothermal–chemotherapy. PTX: Paclitaxel; GNR: Gold NanoRods; NIR: Near InfraRed. Adapted with permission from [62]. Elsevier, [2016].

To date, PF127 injectable hydrogels (Table 2) have been proposed as delivery vehicles for drug and drug crystals in the treatment of both blood and solid tumors [58,59]. Interestingly, such systems were found to reverse the multi-drug resistance in MCF-7/ADR cells because of the ability to increase the intracellular drug concentration escaping the efflux pumps on the cell membrane [59]. To extend the drug release profiles overtime, nanoparticle carriers (e.g., micelles or polymeric nanoparticles) loaded with the cytotoxic agent were incorporated into the hydrogels [60,61,62]. This approach allowed a co-delivery of 5-Fluoruracil (5-FU) and Doxorubicin-loaded Poly(d,l-lactide-*co*-glycolide) nanoparticles (DOX@PLGA) for the in vitro and in vivo treatment of melanoma [61]. When metal nanoparticles (e.g., Cu or Au) were used as loaded nanocarriers, photothermal and hyperthermia effects were achieved (Figure 4) [62,63]. 

Despite the advantageous features of poloxamers, these polymers suffer from weak mucoadhesivity, poor mechanical properties, and short residence time due to the easily dissolution at the action site [64]. To overcome these drawbacks, PF127 was mixed with different polymers from synthetic (polyacrylic acid (PAA) or α-Tocopheryl Polyethylene glycol 1000 Succinate (TPGS)) [65,66] or natural (Hyaluronic acid (HA)) [67,68] origin to increase the gel strength [65] and enhance the drug efficiency [66]. Finally, it should be cited the incorporation of cyclodextrins (α-CD) into the hydrogel network for the preparation of effective depot system in cervix and breast cancer treatment [69]. A further improvement consisted in the insertion of graphene oxide (GO) or reduced graphene oxide (rGO) materials, with the formation of hybrid hydrogels with more sustained drug delivery behavior [70].

### 2.3. Polyesters

During the last decades, thermosensitive in-situ gels of amphiphilic copolymers based on biodegradable polyesters and polyethylene glycol (PEG) have represented a suitable alternative in the intratumoral delivery of hydrophobic therapeutics [71], allowing to recover high drug concentration at the tumor site while overcoming, at same time, the limitations usually associated with the systemic administration of these drugs [72]. The advantages of this class of polymers arise from the possibility to ensure both a physical targeting to the cancer site and a controlled/sustained delivery of hydrophobic drugs [73], as well as from their high biodegradability which allows the obtainment of stimuli responsive and biocompatible delivery platforms [74]. On the other hand, the main drawback of such materials is that their acidic degradation by-products significantly influence the pH value of the surrounding media, with potential limitations in biomedical applications [75]. 

**Figure 5:**
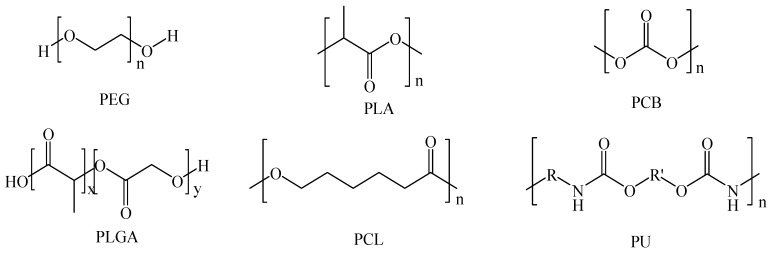
Schematic representation of poly(ethylene glycol) (PEG) and main biodegradable polyesters. PLA: Polylactide; PCB: Polycarbonate; PLGA: Poly(lactide-*co*-glycolide); PCL: Poly(*ε*-caprolactone); PU: Poly(urethane).

Different biodegradable polymers have been proposed for the development of injectable hydrogels, each showing peculiar features and biological performances (Table 3). The structures of the main polyesters employed to this regard are sketched in Figure 5.

Biodegradable poly(d,l-lactide)-poly(ethylene glycol)-poly(d,l-lactide) (PLA-PEG-PLA) amphiphilic triblock copolymer showed the ability to self-assembly in aqueous medium into core-shell micelles, forming a physical network when exposed to the body temperature [76]. Injectable thermo-sensitive PLA–PEG–PLA for the local delivery of Gemcitabile (GEM) and Cisplatin (CisPt) was employed to promote synergistic combination therapy against pancreatic cancer [77]. Alternatively, poly(d,l-lactide) PLA was combined with pluronic L (PL) moieties in the preparation of three-block hydrogels (PLA–PL–PLA) proposed for intraperitoneal therapy of colon cancer [78,79]. This amphiphilic copolymer displayed thermosensitive behavior freely flowing at lower temperatures but turning into gel at body temperature. d,l-lactic (LA) acid oligomer combined with methoxy poly(ethylene glycol) and poly(octadecanedioic anhydride) was employed in the preparation of thermosensitive amphiphilic triblock copolymer suitable for local cancer chemotherapy. In particular, paclitaxel (PTX) loaded into LA oligomer nanoparticles could be stored as freeze-dried powders, and easily re-dispersed into aqueous medium at ambient temperature, forming a hydrogel in the injection site [80].

**Figure 6:**
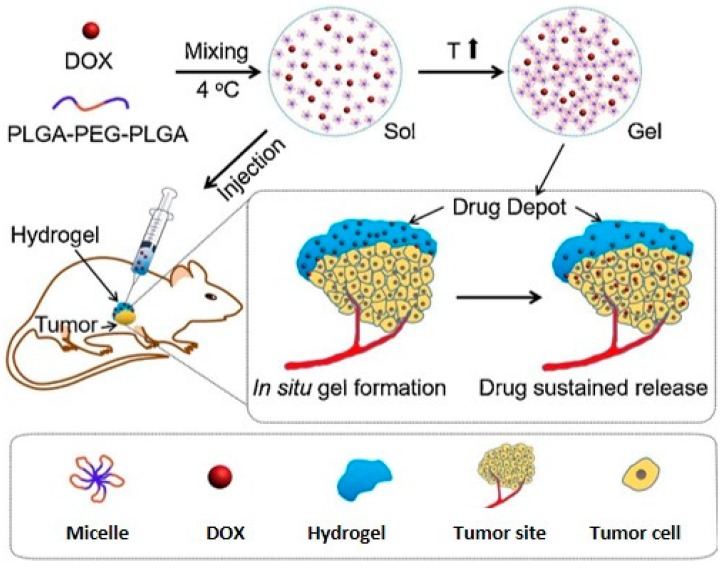
Schematic illustration of localized hydrogel formation and drug release. Adapted with permission from [86]; Elsevier, [2018].

Poly(d,l-lactide-*co*-glycolide) (PLGA) and PEG triblock copolymer (PLGA–PEG–PLGA) hydrogels were synthesized via ring-opening polymerization of d,l-lactide (LA) and glycolide (GA) in the presence of PEG and Tin (II) 2-ethylhexanoate as macroinitiator and catalyst, respectively. Thermo-induced gelation of amphiphilic PLGA–PEG–PLGA can be related to the micellar aggregation as a consequence of the increase in the hydrophobic interactions between the PLGA moieties and the partial dehydration of the PEG chains [81,82]. Literature data indicates that the transition temperatures of PLGA–PEG–PLGA gels were in the range 10–40 °C for a polymer concentration of 15-20% wt [83]. Copolymer concentration influenced sol–gel transition temperature, because the formation of the micellar aggregation network was simplified when the concentration of the polymer increased [84]. PLGA–PEG–PLGA gel was proposed as a carrier of topotecan (TPC), DOX, CisPt, and methotrexate (MTX), and employed for the treatment of osteosarcoma in in vivo experiments (Figure 6) [85,86]. 

Injectable thermosensitive hydrogel can be loaded with either drug or drug-loaded nanoparticles [87]. In particular, the interaction of ionic drugs with specific surfactants has been exploited to achieve sustained release of 2-methoxyestradiol (ME) and Cytarabine (CYT) in the therapy against leukemia and breast cancer, respectively [88,89]. Additionally, drug-loaded particles entrapped in a PLGA–PEG–PLGA hydrogel have been proposed as dual-stimuli responsive drug delivery systems combining the pH-responsivity of the nanoparticles with the temperature response of the PEGylated polyester gels [90,91]. In addition, in a modern scheduled treatment, sustained co-delivery of DOX and sRNA@Poly(ethyleneimine)-Lysine (PEI-Lys) complexes displayed significant synergistic effects in promoting the PLK1 silencing, tumor apoptosis, and cell cycle regulation of osteosarcoma cells [92]. 

In the pharmaceutical and biomedical fields, the sustained release of both hydrophobic and hydrophilic drugs from a single release device represents a newsworthy challenge, exhibiting different clinical survival advantages compared with the single drug treatment. To this regard, a strategy to realize the synchronous, sustained co-delivery of hydrophilic CisPt and hydrophobic PTX in one injectable device was achieved by synthesis of a Pt(IV) prodrug based on MPEG–PLGA, able to self-assemble in a core-corona micelle showing hydrophobic inner cores where PTX can be incorporated [93]. 

Finally, a promising strategy involved the use of cytokine-carrying thermosensitive MethoxyPEG (MPEG)−PLGA hydrogels followed by injection of vaccine vectors loading antigens [94]. This device provides a sustained release profile of granulocyte-macrophage colony-stimulating factor, able to facilitate proliferation, recruitment, and maturation of dendritic cells and macrophages at the site of inoculation, providing an efficient tool proposed in the melanoma therapy. 

ε-Caprolactone was employed in the synthesis of amphiphilic block copolymers bearing PEG pendants. Different injectable Poly(*ε*-caprolactone) (PCL)-based nanocomposite hydrogels with multicomponent compatibility were proposed for the sustainable release of therapeutics, such as PTX, Camptotechin (CPT), 5-FU, and DOX. Three-block copolymers (PEG–PCL–PEG) were prepared by ring-opening polymerization in presence of Tin(II) 2-ethylhexanoate as macroinitiator [73,95,96,97]. Alternatively, PCL–PEG diblock [98] and PCL–PEG–PCL copolymers [99,100,101,102] were synthesized in the presence of 1,4,8-trioxa[4.6]spiro-9-undecanone to obtain a modified PCL able to undergo PEGylation reaction.

A MPEG–*b*–PCL copolymer diblock was proposed in the synthesis of supramolecular hydrogels by combination with α-CD to achieve an injectable delivery system for the release of PTX, DOX, and CisPt in lung and bladder tumors [32,103]. In these systems, α-CD were selectively inserted onto the linear polymer chains, and the resulted supramolecular complex aggregated in packed columns, mainly formed by host–guest interactions or π–π stacking between polymeric chains [104]. These systems have attracted special interest because of their favorable properties, such as thixotropy and reversibility, with their in situ encapsulation characteristics able to prolong the retention time in cancers, reducing side effects [105]. In another system, the coordination between platinum(II) atoms and carboxylic groups of poly-(acrylic acid) (PAA) blocks induced poly(ethylene glycol)–*b*–poly-(acrylic acid) (PEG–*b*–PAA) self-assembly into micelles, with the supramolecular hydrogels eventually formed by the addition of α-CD [106]. Different supramolecular hydrogels based on PEG block polymers (e.g., nucleobase (adenine/thymine)-terminated PEG) were tested for the buccal delivery of DOX in in vivo mouse models [107]. Folic acid (FA)-modified cationic and amphiphilic MPEG–PCL–PEI–FA was proposed as supramolecular system able to form polyplexes with anionic plasmid for sustained gene delivery effectively inhibiting in vivo tumor growth [108].

Drug delivery systems based on PEG–PCL–PEG were loaded with 5-FU and PXT and tested in in vivo experiments for the treatment of colon and breast tumors, respectively [73,95]. Another promising injectable hydrogel for in situ gel-forming controlled drug delivery systems is based on PCL–PEG–PCL, due to several benefits, such as prolonged drug release, sol–gel transition around the body temperature, and ease of handling, being in a solid state at room temperature [109]. In situ gelling materials based on PCL–PEG–PCL loaded with PTX and CPT were proposed as drug delivery systems against breast and gastro-intestinal cancers, with excellent results in both in vivo and in vitro experiments [96,97]. However, the preparation of the anticancer-gel formulations require high temperatures or extended times, which are unsuitable for formulations containing unstable drugs [110]. Moreover, strong hydrophobicity and high crystallinity of PCL units confer to PCL–PEG–PCL a slow degradation rate, which is not always desirable.

To address this concern, chemical modification of PCL allowed the synthesis of new polymeric systems with improved properties. In particular, PCL modified with cyclic ether pendant groups, i.e., poly(*ε*-caprolactone-*co*-1,4,8-trioxa[4.6]spiro-9-undecanone)-poly(ethyleneglycol)-poly(*ε*-capro- lactone-*co*-1,4,8-trioxa[4.6]spiro-9-undeca-none), were prepared [111]. The insertion of cyclic ether pendant groups into PCL units was performed by copolymerization of 1,4,8-trioxa[4.6]spiro-9-undecanone with PCL, and the resulting macromer showed modified gelation performances as a consequence of the changing of PCL crystallization properties. By this approach, injectable carriers for DOX and PXT were obtained and proposed for the treatment of breast and liver cancers [99,100,101,112].

Methoxy poly(ethylene glycol)–*b*–poly(*ε*-caprolactone-*co*-1,4,8-trioxa[4.6]spiro-9-undecanone) (PEG–PCL) diblock copolymer was employed to prepare host–guest inclusion injectable nanocomposite devices based on surface-modified gold nanorods, PTX/PEG–PCL nanoparticles, and α-cyclodextrin [98]. A single local injection of this hydrogel allowed to deliver abundant PTX/PEG–PCL nanoparticles and gold nanorods at the target site, developing remarkable anticancer activity and photothermal effect. Alternatively, the coupling of PTX/PEG–PCL with α-CD allowed the synthesis of supramolecular hydrogels based on the hydrophobic aggregation of pseudorotaxane between cyclodextrins and block copolymers [113].

Co-delivery of anticancer agents and radiosensitizer isotopes was exploited in the design of innovative drug delivery systems able to combine the effects of chemo- and radio-therapy with reduction of the damage to normal tissue and improved therapeutic efficiency [114]. Specifically, PEG–PCL-based hydrogels were employed in the preparation of multifunctional devices for the delivery of DOX and β-emitter species, such as iodine-131 and rhenium-188, for the treatment of the hepatocellular carcinoma [102,115]. Finally, an advanced system involving linear copolymer formed by poly(*ε*-caprolactone) was proposed for transcatheter arterial chemoembolization, a technique based on the combination of chemotherapeutic efficacy from delivered anticancer drugs and a blockage of tumor feeding vessels with an embolic material [116]. Specifically, sulfamethazine-based anionic pH-sensitive block PCL copolymer was fabricated by free radical polymerization [117]. Aqueous solutions of the synthesized copolymer underwent a sol-to-gel phase transition upon lowering the environmental pH, and created a gel region able to cover the physiological conditions and low pH environments typical of the tumor site.

Polyurethane (PU) derivatives, such as poly(amino ester urethane) (PAEU) block copolymers, were employed as drug delivery systems, thanks to their ability to form electrostatic interactions and hydrogen bonds with bioactive molecules, and to exhibit sol–gel phase transition after injection into the body. PAEU copolymers were proposed for the fabrication of injectable radiopaque embolic materials, based on a mixtures of an aqueous copolymer solution and Lipiodol, a commercial long-lasting X-ray contrast agent [118]. In particular, exploiting the influence of pH and temperature on the self-assembly capacity of this polymeric material, a dual drug delivery system was proposed as a carrier for the regional release of DOX in the liver compartment. Additionally, target-specific release of CisPt was proposed by incorporation of CisPt chondroitin sulfate-based nanogels into pH- and temperature-responsive PEG–PAEU hydrogels [119]. In this case, ionic interactions, under physiological conditions, between the tertiary amine and sulfate groups allowed to form hydrogel networks able to selectively bind a receptor specifically expressed on cancer cells [120].

Linear copolymers obtained by suitable mixing of polyester monomers were used to synthesize injectable hydrogels with tailored properties due to their specific hydrophobic/hydrophilic balance. To this regards, PCLA–PEG–PCLA triblock copolymer was synthesized using a ring-opening copolymerization involving ε-Caprolactone and LA, in the presence of PEG and Tin(II) 2-ethylhexanoate. In particular, amphiphilic copolymer was conjugated with heparin to construct non-anticoagulant heparin prodrugs loaded in thermosensitive hydrogel for anti-metastasis treatment [121] and as a GEM carrier for the treatment of pancreatic cancer [122]. Moreover, PCLA–PEG–PCLA copolymer was modified via polyaddition polymerization with sulfamethazine, acting as anionic pH-sensitive moiety, to synthesize a dual stimuli responsive polymeric system, proposed for the DOX release in liver cancer [123]. Finally, injectable pentablock copolymer hydrogels PEG–PCL–PLA–PCL–PEG, with different ratios of PCL and PLA, were proposed as single-shot sustained release of vaccines. Specifically, vaccine was encapsulated into PLGA nanoparticles and incorporated in the thermoresponsive hydrogels in order to modulate gelation temperature and minimize burst release of antigen and adjuvants in the treatment of melanoma [124]. Nevertheless, the synthetic strategies involving lactide, glycolide, or *ε*-caprolactone derivatives to generate a temperature-sensitive and biodegradable polymeric backbone suffered from the lack of chemical functionality in the parent aliphatic polyesters that makes it difficult to modify the polymeric chains.

A valuable alternative way exploited the employment of methyltrimethylcarbonate (PCB), cyclic carbonates derived from 2,2-bis(methylol) propionic acid (bis-MPA), as synthon for functional biodegradable monomers [16]. Ring-opening polymerization, followed by *N*,*N*’-dicyclohexylcarbodiimide-mediated condensation, was the synthetic strategy proposed to prepare hydrophilic/hydrophobic PEG-functionalized cyclic carbonate based on 2,2-bis(methylol)propionic Acid (bis-MPA) [125]. Micellization provided a physical cross-linked system, displaying a lower critical solution temperature at values near the body temperature that can be suitable for PXT release against hepatic cancer cells. A different protocol involved the formation of a biodegradable polymeric biomaterial consisting of PEG and a polycarbonate of dihydroxyacetone (pDHA), proposed for the prevention of the seroma post-operative complications following ablative breast cancer surgery [126]. Vitamins E and D-functionalized polycarbonates were proposed as a hydrophobic block in the synthesis of three-block copolymers able to form physically cross-linked injectable hydrogels for local and sustained delivery of herceptin in breast cancer treatment [127,128].

### 2.4. Polyacrylates

**Figure 7:**
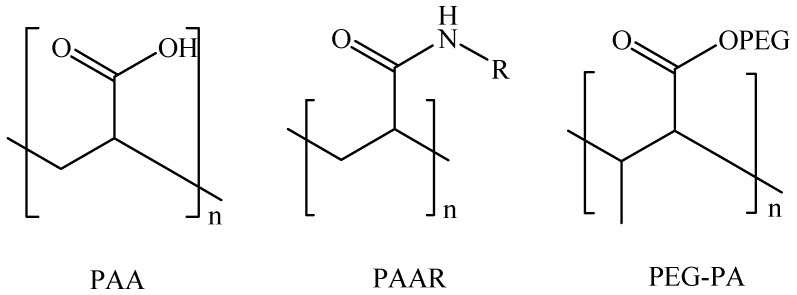
Schematic representation of the main acrylate polymers. PAA: Poly(acrylic acid); PAAR: *N*-alkyl poly(acrylic amide); PEG-PA: PEGylated poly(methacrylic acid).

Photo-induced radical polymerization involving acrylate monomers and/or functionalized macromers represents an alternative to thermal gelation in the preparation of injectable hydrogels able to be self-assemble after injection following a UV-irradiation (Figure 7) [129,130].

The main component of this class of materials enclosed PEG acrylate polymers (PEG-PA), which was designed to allow the insertion of PEG properties (e.g., non-cytotoxicity, non-immunogenicity, and ability to reduce opsonization) within a hydrogel network, showing increased drug loading capability and retention time and improved mechanical properties (Table 4) [23,131]. 

This approach was investigated in the treatment of glioblastoma, employing a system based on polyethylene glycol dimethacrylate (PEGDMA). The photopolymerizable monomer was UV-irradiated in the brain tumor resection bed and employed for the delivery of Temozolomide (TMZ) and Paclitaxel (PTX) [132,133]. This approach could present several advantages, including the killing of the tumor cells that, after the resection of the main primary tumor, could infiltrate the brain tissue and the parenchyma.

Hybrid materials were also prepared by incorporating carbon nanotubes [134] or Zn ferrite nanoparticles [135] for breast cancer treatment by combined DOX/photothermal and thermal ablation therapy, respectively. Injectable hydrogels, proposed for the thermo-responsive delivery of different drug molecules to prostate cancer in vivo, were prepared by radical polymerization of oligo(ethylene glycol) methacrylate (OEGMA) monomers [136]. In another study, PAA was combined with a poly[4-(2,2,6,6-tetramethyl piperidine-*N*-oxyl)aminomethylstyrene]–*b*–poly(ethylene glycol)–*b*–poly[4-(2,2,6,6-tetramethylpiperidine-*N*-oxyl)aminomethylstyrene] (PMNT–PEG–PMNT) triblock copolymer to obtain a redox-active polyion complex for the local protein therapy of murine colon cancer [137].

A different approach involved the synthesis of specific gold nanorods incorporated into the three-dimensional network achieved by radical polymerization of methacrylated poly-*β*-cyclodextrin (MPCD)-based macromer and *N*-isopropylacrylamide (NIPAAm) as a poly(*N*-Alkylacrylamide) (PAAR) derivative [138]. The hydrogel, exhibiting near-infrared and pH responsivity, was efficiently loaded by host–guest interactions with adamantane-modified DOX prodrug, and its efficiency was tested in in vitro tests against MCF7 (breast) and HeLa (cervix) cancer cells, and in in vivo experiments carried out in the treatment of murine sarcoma.

Alternatively, thermoresponsive supramolecular poly(*N*-acryloyl glycinamide-*co*-acrylamide) (PNAm) hydrogels, bearing polydopamine-coated gold nanoparticles and DOX, were fabricated by radical photopolymerization [139], and proposed as a breast filler. This system, after heating in the sol state, was injected into the cavity of resected breasts, where a rapid gelation occurred during cooling to body temperature.

### 2.5. Synthetic Polypeptide

**Figure 8:**
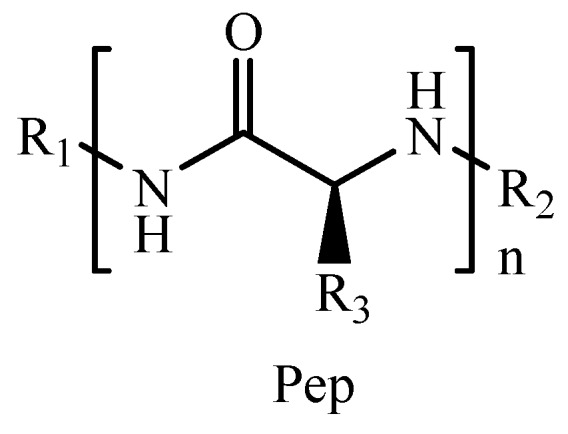
Schematic representation of synthetic polypeptides.

Polypeptides (Pep) are synthetic protein-mimicking materials particularly attractive for their biocompatibility and biodegradability [140,141,142]. Another advantage of this class of compounds lies in the great chemical diversity due to the wide number of monomer sources from 21 natural amino acids and their synthetic derivatives (Figure 8).

**Figure 9:**
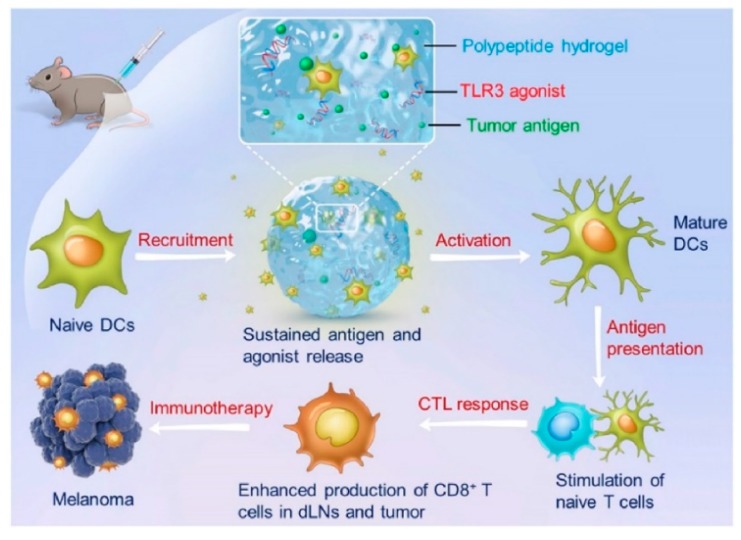
In vivo modulation of dendritic cells (DCs) by sustained release of tumor antigens and tumor cell lysates 3 (TLR3) agonist from a polypeptide hydrogel, evoking a strong cytotoxic T-lymphocyte (CTL) response. With permission from [157]; Elsevier, [2018].

In addition, exploiting intramolecular hydrogen bonds within peptide backbones, polypeptides can adopt ordered secondary structures (i.e., *α*-helix and the *β*-sheet) that confer them the self-assembly behavior. Self-assembling polypeptides were employed as starting materials for the preparation of injectable hydrogels (Table 5) [143,144,145] via gelation processes of their aqueous solutions upon changes in pH, ionic strength, or temperature. The introduction of cytotoxic molecules into the solution led to the encapsulation of bioactive agents for the treatment of different tumors. In detail, ionic gelation was proposed for the preparation of Ce6 carrier system [146] for breast cancer, and stimulation of immune system in health mice [147,148]. Thermo gelation processes were used for the fabrication of injectable hydrogels for TMP-2 [149] and DOX-based therapy [150,151] of breast, cervix, and lung cancers, as well as for DOX or gene (CDN) administration with simultaneous stimulation of immune responses [152,153]. Furthermore, DOX@Liposome formulations were loaded in Pep hydrogels for an Losartan (LST) combination therapy [154]. Another approach for the preparation of starting materials for injectable hydrogels involves the conjugation of peptide moieties to oligoethylene glycol (OEG) [141] or PEG derivatives, with the formation of PEGylated [155,156,157,158] or block [15,159,160] copolymers. Such hydrogels were found to be suitable for the preparation of pro-drugs [160,161,162] and the delivery of different clinically relevant cytotoxic agents, with the possibility to trigger the releasing profile in response to physiological stimuli such as pH [155], temperature [158], and cell redox state [15,160,161,162], or stimulate the immune system (Figure 9) [157]. 

Disulfide bonds were also employed for the preparation of thermo-responsive injectable hydrogels. For example, PEGylated disulfide bond containing poly(l-cysteine) derivative (poly(l-EGx-SS-Cys)) possessed an irreversible thermo-responsive behavior in water, probably ascribed to chemical cross-linking caused by disulfide bond exchange. A thermogel consisting of PEG and poly(l-EG4-SS-Cys) diblock copolymer was used as reduction responsive injectable hydrogel [164]. Physical cross-linking approach was also employed for the preparation of injectable hydrogels with excellent shearing thinning features using PEG_44_-NH_2_ as a macroinitiator [165].

### 2.6. Dendrimers and Other Systems

**Figure 10:**
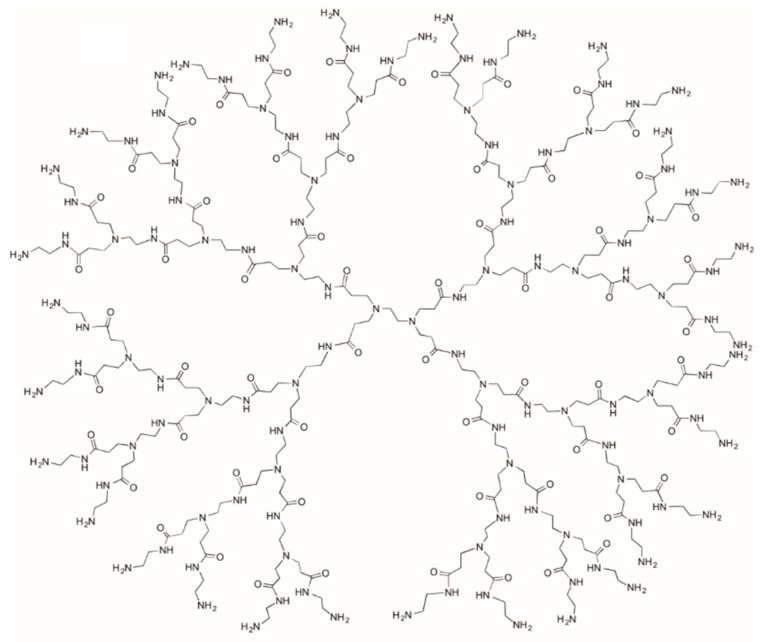
Schematic representation of polyamidoamine (PAMAM )dendrimer.

Dendrimers are synthetic branched polymers with a globular structure, nanometric size, and low polydispersity index [166], fabricated via a sequence of reaction steps in which monomer units are added to a Generation 0 core [167]. This class of materials possesses unique features for drug delivery applications, including the high affinity of the inner hydrophobic environment for different drug molecules, the wide number of functional groups suitable for tailored functionalization [167], and the ability to cross the cell membrane via paracellular and endocytosis pathways [168,169]. Different injectable hydrogels based on dendrimers have been proposed in the literature for the treatment of solid cancers (Table 6), mainly consisting in modified PEG [170], poly(amine-ester) [171], and polyamidoamine (PAMAM) (Figure 10) [172,173]. 

The main component of this class of materials enclosed PEG acrylate polymers (PEG-PA), which was designed to allow the insertion of PEG properties (e.g., non-cytotoxicity). PEG dendrimers were modified by insertion of disulfide bonds [174,175] or boronic acid moieties [176] to confer redox and pH responsivity, respectively. Boronic acid derivatives were also proposed to enhance the pH biodegradability patterns of injectable hydrogels employed for breast cancer treatment in mouse models [177], while the formation of Shiff’s base with poly-L-lysine (PL) carried out to an effective MTF and 5-FU delivery system to colon C26 cells [34]. Targeting behavior can be conferred by derivatization with heparin residues [178]. PEGylated PAMAM injectable hydrogels with increased solubility and improved biodistribution characteristics [179] were tested as 5-FU carriers or as pH and redox responsive DOX delivery vehicles for head/neck and cervix cancer treatment, respectively [172,173]. Other examples of injectable hydrogels for cancer therapy consist in lipid nanocapsule-based hydrogels able to cross the blood–brain barrier [180], and in pH responsive PVA/GO hybrids loaded with a CPT-CD complex [181]. The latter systems take the advantages of the peculiar properties of the high biocompatible carbon nanostructures [182,183,184].

## 3. Natural Polymers

### 3.1. Polysaccharides

Polysaccharides are widely employed for the fabrication of injectable hydrogels, owing to their outstanding advantages consisting in water affinity, biocompatibility, biodegradability, non-immunogenicity, and non-fouling features. Furthermore, the presence of multiple chemical functionalities (e.g., acid, amine, hydroxyl, and aldehyde groups) allows easy chemical modifications with the obtainment of a plethora of biomedical devices. They exert biological activities such as cell recruiting, cell adhesion, and modulation of the inflammatory process, and the pharmacokinetic profiles can be tailored by choosing the appropriate molecular weight distribution [185,186].

Polysaccharides are obtained from renewable plant and animal sources, including algae (e.g., dextran, alginate), plants (e.g., cellulose, agarose), microbes (e.g., dextran, gellan gum), and animals (e.g., hyaluronic acid, chitosan). In this review, when polysaccharides are mixed with synthetic polymers to further modify their physical, mechanical, and chemical properties, the resulting systems are referred as N/S hybrids.

**Figure 11:**
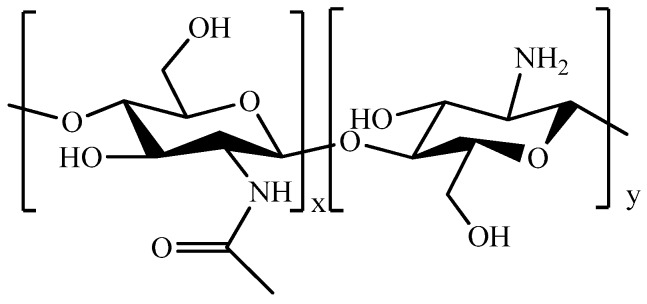
Schematic representation of chitosan (CS).

Chitosan (CS, Figure 11), the *N*-deacetylated derivative of chitin, is a biomaterial with a wide range of biomedical applications due to its high biocompatibility and biodegradability.

In addition, the wound-healing, anti-tumor, and antimicrobial activities, make CS an ideal starting material for designing pharmaceutical injectable formulations (Table 7) [187,188,189]. A CS prodrug of a photosensitizing agent was used as base material to obtain an injectable pH-responsive hydrogel to be used in breast cancer and melanoma therapy [190], whereas the chemical cross-linking of CS with β-GP was proposed in several research works as a valuable strategy to obtain thermo-responsive materials for the treatment of a number of cancer diseases. In more detail, CS/β-GP systems were either employed as platforms for the release of antineoplastic drugs [191,192,193,194] or loaded with nanoparticles bearing the anticancer agent, in order to obtain a more sustained drug release in the site of interest [195,196,197,198]. Other applications involved the possibility to combine chemo- and radio-therapy [195,199], and produce local hyperthermia for different types of cancer [200,201,202]. Thermal gelation of CS in the presence of G carried out to injectable hydrogels for the treatment of breast cancer [203], while mixed polysaccharide hydrogels, including CS-ALG [204] and CS-HA-NIPAAm [205,206] complexes, were designed to produce targeted delivery of anti-VEGF antibody [204], as well as pH-responsive systems for the DOX [205] and DOX@GO [206] vectorization to colon and breast cancer, respectively. Injectable hydrogels were also prepared using CS hydrophilic derivatives [207]; for example, CS modified with glycol moieties was covalently linked with PEG to obtain hydrogel materials for the release of self-healing [208] and photosensitizing [209] agents. In another approach, DOX@PLGA nanoparticles were inserted into the hydrogel structure, together with magnetic nanoparticles, to raise a more sustained release profile combined with magnetic ablation of breast cancer [210]. Furthermore, supramolecular hydrogels composed of GCS, PF127, and α-CD were proposed as DOX delivery platforms in the treatment of liver carcinomas [211]. Different modifications involved the bonding of hydroxybutyl [212], hydroxypropyl [7], carboxymethyl [213,214], and carboxyethyl [215,216,217] groups.

In more detais, carboxymethyl chitosan (CMCS) was copolymerized with NIPAAm [213] to obtain pH- and thermo-responsive depots for the on-off release of 5-FU to cervix and breast cancers. hydroxypropyl chitosan (HPCS) was condensed with PPLL dendrimers by Schiff’s bases and subjected to an ionic gelation process in the presence of PEG dendrimers and oxDEX nanoparticles bearing DOX, IL-2, and IFN-γ for a synergistic anticancer therapy.

In another approach, CS [218], PBCS [219], CS-DA [220], or CS alkyl derivatives [214,216,217] were condensed with oxidized polysaccharides, including DEX [218,219], ALG [214,216], HA [217], and PLN [220].

SCS was combined with oxCS [221] or oxALG [222] to obtain pH-responsive injectable hydrogels for DOX sustained release. Other examples of CS derivatives include GTMACS [223] and CS-CAT [224], used for DTX or DOX/DTX combination therapy, respectively. TCS was employed to produce an enzyme-responsive CUR delivery vehicle [226], and CS-TPP was proposed for photothermal therapy in breast and liver cancers [225].

**Figure 12:**
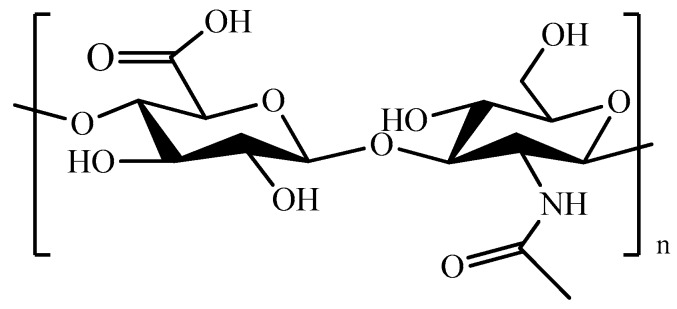
Schematic representation of hyaluronic acid (HA).

Hyaluronic acid (HA, Figure 12), a non-sulfated glycosaminoglycan, is one of the major components of connective tissues and synovial fluid.

**Figure 13:**
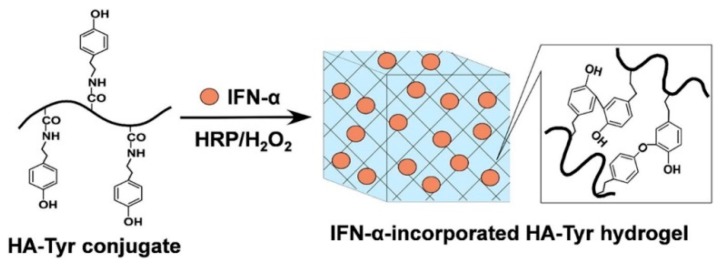
Schematic illustration of in situ formation of IFN-α-incorporated HA–Tyr hydrogels through enzymatic cross-linking reaction. HRP: horseradish peroxidase. With permission from [236]. Elsevier, [2016].

It is able to interact with cell surface receptors (e.g., CD44), thus promoting cell migration, and, in virtue of its high biocompatibility, has been extensively exploited as a starting material for the fabrication of different injectable hydrogel systems (Table 8) [227,228,229,230]. The thermo-gelation of HA in the presence of PF127 carried out to injectable hydrogels suitable for DOX release to breast [231] and colon cancers [68], or for the DOX-DTX synergistic treatment of CT26 cancer cells [67]. Oxidized HA was chemically cross-linked to obtain an injectable biomaterial mimicking embryonic microenvironments, thus exerting and controlling the phenotype of aggressive cancer cells [232]. Injectable HA hydrogels obtained with the same approach were either physically loaded with, or chemically conjugated to, CisPt-loaded HA nanogels for gastric cancer treatment [233]. Different cross-linking strategies involved the preliminary derivatization of HA with Tyr residues [234,235,236], or the insertion of thiol groups [237]. In the first case, horseradish peroxidase (HRP) catalyzed the coupling reaction between HA–Tyr chains with the formation of injectable hydrogels for the delivery of IFN-α to Kidney cancer (Figure 13) [236], while the incorporation of hyaluronidase allowed the selective vectorization of conjugated IFN-α [234] and loaded TZB [235] to liver and breast cancer, respectively.

On the other hand, the oxidation of thiol groups was exploited to generate disulfide bonds acting as cross-links of the hydrogel. The resulting redox-responsive material was employed as a delivery vehicle of DOX and the combinations of DOX–SRB and DOX–SRB–MTF [237]. HA was also employed as a functional element for the enzymatic synthesis of PEGylated dendrimers able to modulate the cellular phenotype of human mammary cancer epithelial cells and mouse myoblasts [240].

Finally, the incorporation of MSNs [238] and α-CD–AuBNs–MSNs [239] within HA hydrogels allowed the fabrication of hybrid systems suitable for photothermal DOX combination therapy of mammary and squamous carcinoma, respectively.

**Figure 14:**
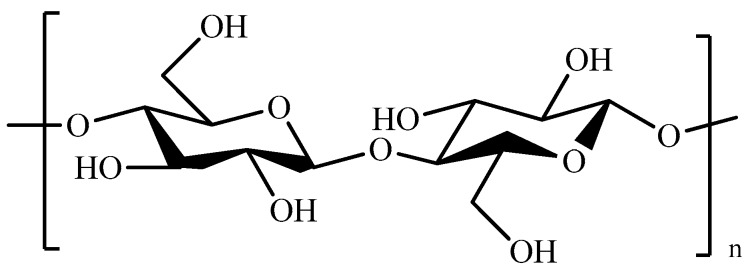
Schematic representation of cellulose (CL).

Cellulose (CL, Figure 14) is a polysaccharide consisting of repeating β-*D*-glucopyranose units obtained from different sources, including wood pulp, cotton, tunicates, fungi, bacteria, and algae [241].

The superior biological features, together with the large availability and low cost, make CL-based materials suitable for a wide range of applications, including biomedicine (Table 9) [242].

Hydrophilic CL derivatives, such as quaternized cellulose [243] and hydroxypropyl methyl cellulose [244], were investigated for the DOX-based and PTX/TMZ therapy of hepatocellular carcinoma [243] and glioma [244], respectively. Pristine CL was also tested as a base material for the fabrication of hybrid hydrogels for the photothermal treatment of melanoma and hepatic cancer, both in vitro and in vivo [245], with black phosphorus nanosheets acting as active agent.

**Figure 15:**
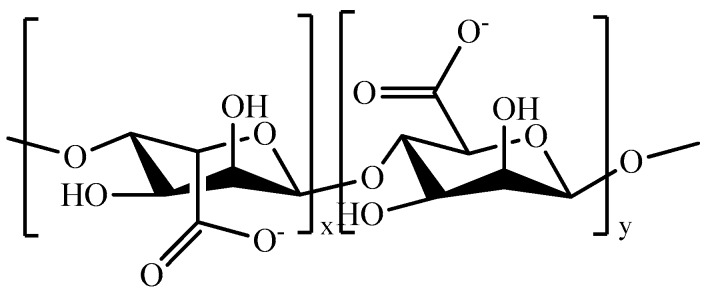
Schematic representation of alginate (ALG).

Alginate (ALG, Figure 15), an anionic biopolymer consisting of units of mannuronic acid and guluronic acid in irregular blocks [246], is widely used in biomedical field due to its several favorable properties, including biocompatibility, hydrophobicity, and availability of hydroxyl and carboxyl groups for tailored chemical modifications (Table 9) [247].

Injectable hydrogels prepared by ionic gelation were proposed for the delivery of CisPt dendrimers to breast and lung cancer cells with high efficiency [248], as well as for the incorporation of magnetic nanoparticles for the thermal ablation of different types of cancers, including breast, ovary, glioblastoma, and colon [201]. The insertion of NIPAAm moieties carried out the formation of thermo-responsive vehicles of gene [249] and DOX@micelles [250] to prostate cancer and osteosarcoma. Further modifications of ALG chains involved the oxidation to aldehyde derivatives, suitable for coupling with PEI polymers. The obtained in situ gelling systems were proposed as delivery systems for core-shell nanoparticles loaded with CisPt and PTX, and found to be effective in the treatment of breast, skin, and liver neoplasia [251,252].

**Figure 16:**
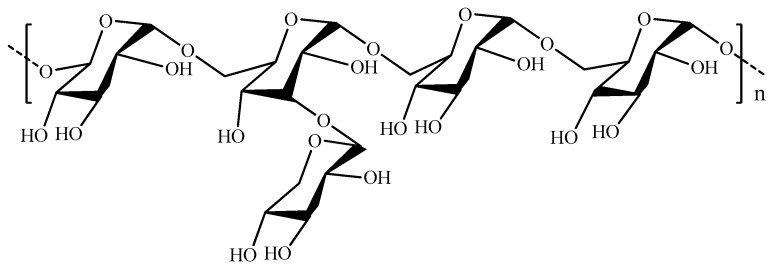
Schematic representation of dextran (DEX).

Dextran (DEX, Figure 16) consists of glucose monomers linked via α-1,6 glycosidic bonds, with branches originating from α-1,3 linkages. It finds a wide range of applications in the biomedical field, due to its high availability, low cost, and easy chemical modification.

Moreover, its high stability, hydrophilicity, absence of toxicity, and biodegradability make this polysaccharide an ideal drug delivery carrier (Table 9) [261]. It is able to promote the penetration of chemotherapeutic agents in tumor masses [262], thus allowing the fabrication of effective delivery vehicles for cancer treatment [263]. Preliminary derivatization of dextran, including oxidation [253,254] and conjugation to acrylic [260] or thiol groups [255], was carried out to obtain effective carriers for the delivery of cytotoxic drugs [253,254], gene [260], or DOX in combination with Bismuth Nanoparticles in a combined X-ray radio- and chemo-therapy [255].

**Figure 17:**
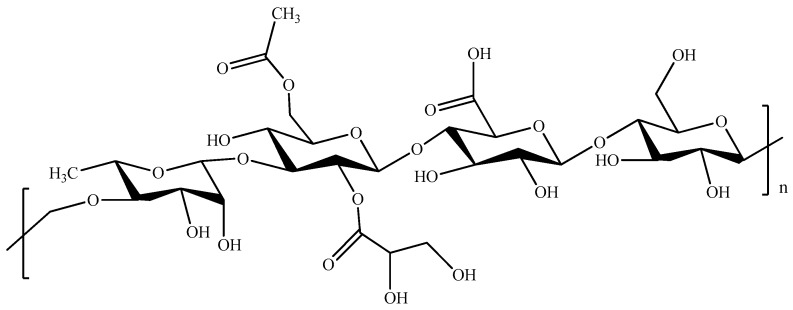
Schematic representation of gellan gum (GG).

Gellan gum (GG, Figure 17) is a linear anionic polysaccharide approved by the FDA as an additive in food and pharmaceutical formulations (Table 9) [264].

Its biodegradability, mucoadhesivity, and thermo-reversible gelling properties make it the ideal candidate for the preparation of injectable matrices to be employed in tissue engineering and wound healing. Injectable nanocomposites, consisting of GG hydrogels incorporating drug-loaded nanoparticles, were proposed for the treatment of different cancer diseases. More closely, PTX-loaded liposomes were loaded on a GG hydrogel matrix and the overall system directly instilled in the urinary bladder [256]; whereas, in another work, DOX-loaded CuS nanoparticles were embedded in GG injectable hydrogels for NIR-triggered chemo-photothermal therapy of breast cancer [257].

**Figure 18:**
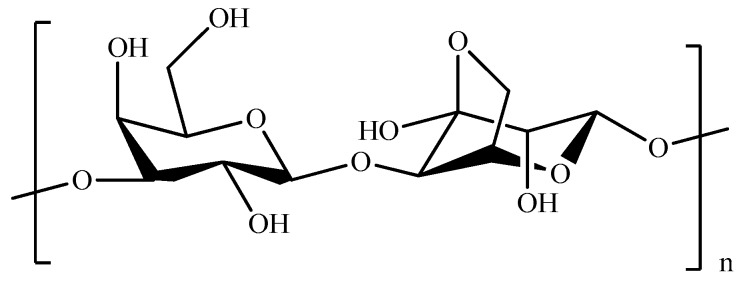
Schematic representation of agarose (AGR).

Agarose (AGR, Figure 18) is an FDA-approved linear polysaccharide derived from marine algae. A robust injectable thermo-responsive AGR hydrogel incorporating sodium humate and DOX was proposed as a valuable tool for chemo-photothermal treatment of breast cancer [258]. Furthermore, DOX@nanoparticles were encapsulated in AGR injectable hydrogels for sustained local drug delivery (Table 9) [259]. 

### 3.2. Proteins

The integration of the structural and functional properties of proteins in injectable hydrogels was also tested, thanks to the high biocompatibility, biodegradability, non-toxicity, and non-immunogenicity of such materials, as well as by virtue of their similarity to naturally occurring components of organs, tissues, and cells (Table 10) [25,265,266,267,268].

Serum albumins, from both bovine and human serum, are the most abundant protein in blood plasma (40–50 mg/mL) and the primary transport proteins of various endogenous and exogenous substances in plasma, including cations, bilirubin, fatty acids, and drugs [269,270].

Albumin from bovine serum (BSA) was proposed as polymeric support in the synthesis of injectable hydrogels for cancer therapy. BSA was added to the cross-linking agent epichlorohydrin to prepare a gel with suitable mechanical strength, viscoelastic behavior, shear thinning, injectability, and self-healing properties useful as DOX delivery vehicles to cervix and breast cancer [269]. Alternatively, an injectable hydrogel consisting of PEG-modified BSA- and PTX-encapsulated red blood cell membrane nanoparticles was proposed to improve the intraperitoneal retention of PTX in the treatment of human gastric cancer [271]. Finally, human serum albumin (HSA) chemically conjugated to PEG dendrimers was suggested as a functional biomaterial for the induction of apoptosis in pancreatic cancer [270].

Gelatin (GEL) represents another interesting protein material able to spontaneously undergo the gel–sol transition process at body temperature. Despite its good biological properties, gelatin hydrogel cannot be used in biomedical applications without chemical modifications, due to its instability under physiological conditions and, also, poor mechanical properties [272]. Different approaches were proposed to improve its performance in the biomedical field [273]: GEL–dendrimer [274], GEL–pectin [275], and GEL–CS [276] composites, cross-linked by means of HRP chemistry [274,275] or ionic gelation [276], were successfully employed in lung and skin carcinomas studies, and for the controlled release of DOX@Liposome. In addition, GEL injectable hydrogels were proposed as DOX carriers in the treatment of prostate cancer [277] in a multifunctional system, also acting as regenerative matrix with pronounced adhesion to abdominal tissue that, by in situ polymerization, allow to overcome the inconvenience usually related to radical prostatectomy. Moreover, due to its surfactant properties [278], GEL was also employed for the fabrication of thermo-responsive hybrid hydrogels for the controlled release of DOX to gastric cancer [279], with improved efficiency due to the incorporation of rod-like-shaped nanoparticles, such as carbon nanotubes [280,281]. Finally, an injectable and colloidal hydrogel composed of amphoteric GEL nanoparticles and polydopamine (PDA) nanoparticles was developed to realize multi-stimuli (pH, enzymes, and near-infrared light)-responsive drug delivery properties and combined chemo-photothermal cancer treatment [282]. Due to the sensitivity of GEL nanoparticles to the tumor microenvironment and PDA nanoparticles to the NIR laser, DOX-loaded hydrogel could show multiple responsivity to acidic pH and NIR laser irradiation, resulting in controlled and sustained anticancer release profiles.

Silk fibroin (SF) was proved to be a biodegradable and biocompatible native natural material derived from Bombyx mori silkworm with safe record in vivo [283,284]. SF hydrogels developed by the protein conformation transition from amorphous to β-sheet induced by physical cross-linking, including the ultrasound assisted processes, possess injectability as well as biocompatibility and safety features [285]. SAL–PTX-loaded silk fibroin hydrogel was fabricated by ultrasound-assisted cross-linkage, without toxic organic solvents and surfactants, for loco-regional tumor treatment and cancer stem cell inhibition in vivo [286]. Additionally, self-assembling pH-responsive silk nanofiber hydrogels with thixotropic properties were proposed to support the injectable delivery DOX for the treatment of breast cancers in mouse models [287]. The possibility to obtain benefits from a photothermal treatment was exploited in the synthesis of SF nanofiber hydrogel systems complexed with lanthanide-doped rare-earth up-conversion nanoparticles and nano-graphene oxide for breast cancer treatment [288]. In this case, a synergistic effect of combined up-conversion luminescence imaging diagnosis and photothermal therapy was confirmed to decrease dosage-limiting toxicity and tissue damage by over-heating and improve the therapeutic efficiency. An innovative approach that drastically reduces gelation times involved an enzyme-mediated cross-linking strategy to produce fast-gelled SF-based injectable hydrogels at physiological conditions [289].

Finally, silk–elastin-like protein (SELP), genetically engineered materials composed of tandem repeats of a six amino acid sequence commonly found in silkworm silk fibroin and a five amino acid sequence commonly found in mammalian elastin, was proposed in the synthesis of injectable hydrogels. This combination of silk and elastin molecular properties results in a polymer which is responsive to temperature increases and irreversibly forms hydrogels at physiological temperature. Gelation occurs without the need of chemically-induced cross-linking, because this phase transition spontaneously occurs when elastin-like units collapse thermodynamically aligning the silk-like units that form hydrogen-bonded beta sheets, and results in a physically cross-linked matrix. SELP-based carriers were applied as a platform for drug delivery with negligible toxicity for the radiation treatment of prostate and pancreas cancers [288,290], for localized delivery in transarterial chemoembolization to treat intermediate stage hepatocellular carcinoma [290,291], or as gene-directed enzyme prodrug therapy [292]. In particular, injectable brachytherapy polymers [290,291] composed of SELP labeled with the radionuclide ^131^I exhibit a gelling transition as a result of two independent mechanisms, firstly involving SELP moieties that, at the body temperature, are rapidly converted into an insoluble material. Afterwards, the high energy β-emissions of ^131^I further stabilize the depot by introducing cross-links within the SELP depot over 24 h. Additionally, SELP-based hydrogel was proposed to overcome the limitations usually associated with the commercial embolic liquids that discourage their employment in transarterial chemoembolization. To this regard, DOX and SRB, two chemotherapeutics used in the treatment of hepatic carcinoma, were incorporated into the in situ gelling liquid embolic composed of SELP polymer [290,291]. Due to its pore size and in vivo gelation properties, SELP restricts the distribution and controls the release of therapeutic viruses, such as herpes simplex virus, for up to one month, representing a valuable approach which may also have significant potential for increasing the safety of adenoviral gene delivery, while not sacrificing efficacy is spatial and temporal delivery of viruses following injection into a localized area [292]. In this way, gene expression levels at the site of interest were localized, prolonged, and significantly increased.

## 4. Conclusions and Future Perspectives

Hydrogel systems represent a relevant class of healthcare products with applications ranging from tissue engineering, bio-sensing, and bio-imaging, to drug delivery [293]. The huge interest in hydrogels is underlined by the worldwide market, estimated at around US$10 billion in 2017 and expected to grow up to US$15 billion by 2020 [294]. Injectable hydrogels have been proved to be a valuable tool for the delivery of anticancer drugs, providing temporal and spatial control over the releasing rate, thus improving the therapeutic index of commonly used chemotherapeutics [29]. To date, a few products are currently available on the market, including CS/Organophosphate (BST-Gel ^®^), PLGA–PEG–PLGA (ReGel ^®^), Poloxamer 407 (LeGOO ^®^), Poly(vinyl methyl ether co maleic anhydride) (Gantrez ^®^) hydrogels, available as cartilage repair [295] hydrogel market, tumors [296], vascular injury [297], and vaccine adjuvants [298].

The main limiting issues, concerning sterilization, scale-up, shelf-life, and user compliance (professional and/or patient), must be addressed before the benefits afforded by injectable hydrogels can be translated into clinical practice. Some formulations are currently in clinical trials, mainly consisting in radiopaque PEG hydrogels (TraceIT ^®^ and SpaceOAR ^®^) useful to improve the target definition of radiotherapy, thus reducing the radiation doses [299,300].

The scientific community recognizes great potential to the use of injectable systems for anticancer delivery, but to definitely replace the conventional therapies with the injectable systems, continuous innovation in the development of new architectures and design strategies is required. For a more effective translation of injectable hydrogels from research into clinical reality, future attempts should be done to explore the possibility of combining chemotherapy, hyperthermia therapy, immunotherapy, and radiotherapy, by selecting appropriate materials and evaluating the biological effects on metabolic and cellular mechanisms, both in the normal and diseased states.

## Figures and Tables

**Table 1:** Composition and anticancer performance of injectable hydrogels based on polyphosphazenes.

Ref	Composition		Carrier Properties		Delivery Properties		Cancer Model			Health Model	
	Hydrogel (Gelation Process)	Composite Component	Degradation Time (Days)	Smart Responsivity	Bioactive Agent (DL% *w/w*)	Release Time (Days)	Type	In Vitro	In Vivo	In Vitro	In Vivo
[40]	PPZ (Physical – T)	- - -	30	- - -	ME (0.15)	35	Breast	MDA-MB-231	MDA-MB-231	- - -	- - -
[44]	PPZ (Physical – T)	- - -	- - -	- - -	DOX (10)	30	Stomach	HSC44Luc	HSC44Luc	- - -	- - -
[45]	PPZ (Physical – T)	- - -	>50	- - -	DOX (0.3–0.6)	35	Stomach	SNU-601	SNU-601	- - -	- - -
[46]	PPZ (Physical – T)	- - -	- - -	- - -	DTX (10)	28#	Stomach	44As3Luc	44As3Luc	- - -	Mice
[47]	PPZ (Physical – T)	- - -	10–20	- - -	DTX (1–3)	10–20	Stomach	SNU-601	SNU-601	- - -	Mice
							Pancreas	AsPC-1	- - -		
							Liver	SNU-398	- - -		
[48]	PPZ (Physical – T)	- - -	- - -	- - -	PTX (0.6–0.9)	60	Colon	DLD-1	- - -	- - -	- - -
							Stomach	SNU-601	SNU-601		
[49]	PPZ (Physical – T)	- - -	- - -	- - -	PTX-DOX (0.6)	60–100#	Stomach	SNU-601	SNU-601	- - -	- - -
[50]	PPZ (Physical – T)	- - -	- - -	- - -	sRNA	30#	Prostate	PC3	PC3	- - -	- - -
[51]	PPZ (Physical – T)	- - -	12–25	- - -	CPT* (10)	60	Lung	A549	- - -	- - -	- - -
							Colon	DLD-1	HT-29		
								HCT-116			
								HT-29			
[52]	PPZ (Physical – T)	CoFe_2_O_4_	30	Magnetic	SN-38 (0.8–0.12)	60	Glioblastoma	U-87	U87	3T3	- - -
[53]	PPZ (Physical – T)	Zn_0.47_Mn_0.53_Fe_2_O_4_	25	Magnetic	- - -	- - -	Glioblastoma	U-87	U87	3T3	Mice

* Conjugated to hydrogel; # from in vivo experiments; DL: Drug loading; T: Temperature; CPT: Camptotechin; DOX: Doxorubicin; DTX: Docetaxel; ME: 2-Methoxyestradiol; PPZ: Poly(organophosphazene); PTX: Paclitaxel; SN-38: 7-ethyl-10-hydroxycamptothecin.

**Table 2:** Composition and anticancer performance of injectable hydrogels based on poloxamers.

Ref	Composition		Carrier Properties		Delivery Properties		Cancer Model			Health Model	
	Hydrogel (Gelation Process)	Composite Component	Degradation Time (Days)	Smart Responsivity	Bioactive Agent (DL% *w/w*)	Release Time (Days)	Type	In Vitro	In Vivo	In Vitro	In Vivo
[58]	PF127 (Physical – T)	- - -	- - -	pH	MLX (7.5)	1	Leukemia	K562	- - -	- - -	- - -
								HL60			
[59]	PF127 (Physical – T)	- - -	- - -	- - -	PTX (2.0) LAP (4.0)	4	Breast	MCF7	- - -	BT 474	Mice
								MCF7-ADR			
[60]	PF127 (Physical – T)	MPEG-PCL	- - -	- - -	Q* (7.0)	9	Ovary	SK-OV-3	SK-OV-3	- - -	- - -
[61]	PF127 (Physical – T)	PLGA	- - -	- - -	5-FU (2.0) DOX* (2.0)	35	Melanoma	B16F10	B16F10	- - -	- - -
[62]	PF127 (Physical – T)	OCS MPEG-AuNRs		- - -	PTX* (34.8)	18#	Liver	HepG2	HepG2	- - -	- - -
[63]	PF127 (Physical – T)	PVP	- - -	- - -	Cu_2_MnS_2_*	- - -	Murine breast	4T1	4T1	- - -	- - -
[65]	PF127/PAA (Physical – I,T)	- - -	- - -	- - -	OXA (2.3)	1	Colon	SW480	- - -	IEC-6	- - -
[66]	PF127/TPGS (Physical – T)	- - -	- - -	Temperature	DTX (5.0)	3	Liver	SMMC- 7721/RT	SMMC- 7721/RT	- - -	- - -
[69]	PF127/β-CD (Physical – T)	- - -	- - -	Temperature pH	CUR (10)	2	Cervix	HeLa	- - -	L929	- - -
							Breast	MCF-7			
[70]	PF127/α-CD (Physical – T)	GO	8	- - -	DOX (6.0) CPT (14)	8 6	- - -	- - -	- - -	- - -	- - -
	PF127/α-CD (Physical – T)	rGO									

* Loaded into composite component; # from in vivo experiments; DL: Drug loading; I: Ionic; T: Temperature; 5-FU: 5-Fluoruracil; CD: Cyclodextrin; AuNRs: Gold nanorods; CPT: Camptotechin; CUR: Curcumin; DOX: Doxorubicin; DTX: Docetaxel; GO: Graphene oxide; rGO: Reduced graphene oxide; LAP: Lapatinib; MLX: Meloxicam; MPEG: Monomethoxy poly(ethylene glycol); OCS: *N-*octyl chitosan; OXA: Oxaliplatin; PAA: Poly(acrylic acid); PCL: Poly(*ε*-caprolactone); PF: Pluronic F; PLGA: Poly(lactide-*co*-glycolide); PTX: Paclitaxel; PVP: Polyvinylpyrrolidone; Q: Quercetin; TPGS: α-Tocopheryl polyethylene glycol 1000 succinate.

**Table 3:** Composition and anticancer performance of injectable hydrogels based on polyesters.

Ref	Composition		Carrier Properties		Delivery Properties		Cancer Model			Health Model	
	Hydrogel (Gelation Process)	Composite Component	Degradation Time (Days)	Smart Responsivity	Bioactive Agent (DL% *w/w*)	Release Time (Days)	Type	In Vitro	In Vitro	In Vitro	In Vitro
[77]	PLA-PEG-PLA (Physical – T)	PLA-PEG-PLA	- - -	- - -	GEM* (10) CisPt* (0.2)	10	Pancreas	Bxpc-3	Bxpc-3	- - -	Mice
[78]	PLA-PL64-PLA (Physical – T)	PLA-PL35-PLA	- - -	- - -	DTX (4.5) LL37* (7.5)	24	Colon	HCT 116	- - -	HEK293	Mice
[79]	PLA-PL35-PLA (Physical – T)	PLA-PL35-PLA	- - -	- - -	OXA DTX* (4.4)	14	Murine colon	CT26	CT26	3T3	- - -
										HEK293	
[80]	MPEG-POA-LAO (Physical – T)	MPEG-POA-LAO	- - -	- - -	PTX* (0.9)	17#	Breast	MCF7	MCF7	- - -	- - -
							Cervix	HeLa	- - -		
[83]	PLGA-PEG-PLGA (Physical – T)	- - -	- - -	- - -	DOX (1.0)	12	Osteosarcoma	Saos-2	Saos-2	L929	- - -
					CisPt (1.0)			MG-63	- - -		
					MTX (1.0)						
[85]	PLGA-PEG-PLGA (Physical – T)	- - -	- - -	- - -	TPT (1.0)	5	Sarcoma	- - -	S180	- - -	- - -
[86]	PLGA-PEG-PLGA (Physical – T)	- - -	44	- - -	DOX (1.0)	15	Osteosarcoma	Saos-2	- - -	- - -	- - -
							Murine osteosarcoma	K7	K7		
[88]	PLGA-PEG-PLGA (Physical – T)	Vesicles	- - -	- - -	CYT* (25.4)	12	Leukemia	K562	- - -	- - -	Rabbit
								HL-60			
[89]	PLGA-PEG-PLGA (Physical – T)	Liposome	- - -	- - -	ME* (5.6)	70	Murine breast	- - -	4T1 mice	- - -	- - -
[90]	PLGA-PEG-PLGA (Physical – T)	Arg Dendrimers	- - -	pH	DOX* (13.6)	20	Murine breast	4T1	4T1 mice	3T3	- - -
		Lys Dendrimers			DOX* (14.3)					RAW267	
[91]	PLGA-PEG-PLGA (Physical – T)	SLN	- - -	pH	ME* (2.05–2.23)	45	- - -	- - -	- - -	- - -	- - -
[92]	PLGA-PEG-PLGA (Physical – T)	PEI-Lys	40	- - -	DOX (1.0) sRNA*	16#	Osteosarcoma	Saos2	Saos2	- - -	- - -
								MG-63	- - -		
[93]	MPEG-PLGA (Physical – T)	MPEG-PLGA	- - -	- - -	PTX (4.0) Pt* (0.8)	80	Ovarian	SKOV-3	SKOV-3	- - -	- - -
[94]	MPEG-PLGA (Physical – T)	- - -	- - -	- - -	GM-CSF (15–25)	14	Murine Melanoma	B16		- - -	- - -
								B16-F10	B16-F10		
[95]	PCL-PEG (Physical – T)	- - -	- - -	- - -	5-FU (1.0)	7	Colon	CT26	CT26	- - -	- - -
[73]	PEG-PCL-PEG (Physical – T)	MPEG-PCL	- - -	- - -	PTX* (8.3)	20	Breast	4T1	4T1	- - -	- - -
[96]	PCL-PEG-PCL (Physical – T)	- - -	- - -	pH	PTX** (20)	30	Liver	HepG2	- - -	L929	Mice
							Breast	MCF7			
[97]	PCL-PEG-PCL (Physical – T)	PCL-PEG-PCL		- - -	CPT* (4.1–13.5)	14	Colon	- - -	CT26	L929	- - -
[98]	MPEG-PCL- α-CD (Physical – T)	MPEG-PCL AuNRs	14 3^§^	- - -	PTX* (3.0)	14	Murine Breast	4T1	4T1	- - -	- - -
[100]	PCL-PEG-PCL (Physical – T)	PCL-PEG-PCL	- - -	- - -	PTX* (1.25, 2.5)	45	Erlich ascites	EAC	EAC	- - -	- - -
							Ovarian	- - -	OVCAR-3		
[101]	PCL-PEG-PCL (Physical – T)	PCL-PEG-PCL	- - -	pH	DOX* (1.0)	35	Liver	HepG2	- - -	- - -	- - -
							Breast	- - -	Bcap-37		
[102]	PCL-PEG-PCL (Physical – T)	HA	- - -	pH	DOX ^131^I* (0.5–2)	35	Liver	HepG2	HepG2	- - -	- - -
[32]	MPEG-PCL-α-CD (Physical)	MPEG-PCL	- - -	- - -	PTX* (18.8)	3	Lung	A549	- - -	- - -	- - -
[103]	MPEG-PCL-α-CD (Physical)	MPEG-PCL	- - -	pH	DOX* (15) CisPt* (20)	8	Bladder	EJ	- - -	HEK293	- - -
[106]	MPEG-PAA-α-CD (Physical)	MPEG-PAA	- - -	- - -	CisPt* (0.5–1.0)	4	Bladder	EJ	- - -	HEK293	- - -
[107]	APEGA/TPEGT-α-CD (Physical)	- - -	- - -	- - -	DOX (0.2–0.6)	11	Buccal	- - -	U14	L929	- - -
[108]	MPEG-PCL-PEI-α-CD (Physical – SE)	- - -	- - -	- - -	PTX (10) pDNA	7#	Liver	HepG2	- - -	HEK293	- - -
							Lymphoma	Bcl-2		LO2	
[112]	PCL-PEG-PCL/ MPEG-PPFEMA (Physical – T)	- - -	- - -	- - -	PTX (4.2) DOX (4.2)	42	Breast	MCF7	Bcap-37	- - -	- - -
[113]	MPEG-PCL-α-CD (Physical)	MPEG-PCL	- - -	- - -	PTX* (1–3)	20	Murine breast	- - -	4T1	- - -	- - -
[115]	PCL-PEG-PCL (Physical – T)	Liposome	- - -	- - -	^188^Re DOX* (2.0)	10	Murine liver	BNL-Luc	BNL-Luc	- - -	- - -
[117]	PCLMA-PEGMA-SMA (Chemical – RP)	- - -	- - -	pH	DOX (0.2–0.4)	27	Liver	HepG2	VX2	L929	- - -
[118]	PAEU (Physical – T, pH)	- - -	- - -	- - -	DOX (10)	14	Liver	HepG2	VX2	L929	- - -
[119]	PEG–PAEU (Physical – T)	CHS-Nanogel	28	pH	CisPt* (0.2)	14	Lung	A549	- - -	3T3	Mice
[121]	PCLA-PEG-PCLA (Physical – T)	PCLA-PEG-PCLA	35	- - -	Hep*	10	Cervix	HeLa	HeLa	HaCaT	- - -
[122]	PCLA-PEG-PCLA (Physical – T)	MMT	56	- - -	GEM* (10)	7	Pancreas	- - -	Panc-1	293T	- - -
[123]	PCLA-PEG-PUSSM (Physical – T)	- - -	28	pH	DOX (5.0)	28	Liver	HepG2	VX2	293T	- - -
[124]	PEG-PCL-PLA-PCL-PEG (Physical – T)	PLGA	30	- - -	OVA (0.8) MPL (0.6) QA (1.1)	30	Melanoma	- - -	B16 OT-I B16 OT-II	- - -	- - -
[125]	Bis-MPA-PEG (Chemical – ROP)	- - -	- - -	Temperature	PTX (3.9)	7	Liver	HepG2	- - -	- - -	- - -
[126]	PEG-pDHA (Physical)	- - -	1	- - -	- - -	- - -	Breast	- - -	Rat	- - -	- - -
[127]	VitE-PCB-VitE (Physical)	- - -	42	- - -	TZB (4.0)	16		MCF-7	- - -	HDF	- - -
								BT474	BT474		
[128]	VitD-PCB-VitD VitE-PCB-VitE (Physical)	- - -	- - -	- - -	TZB (4.0)	42	Breast	BT474	BT474	- - -	Mice

* Loaded into composite component; # from in vivo experiments; DL: Drug loading; I: Ionic; T: Temperature; RP: Radical polymerization; ROP: Ring-opening polymerization; SE: Solvent evaporation; Arg: Arginine; 5-FU: 5-Fluoruracil; CD: Cyclodextrin; APEGA: Adenine-terminated poly(ethylene glycol); AuNRs: Gold nanorods; CHS: Chondroitin sulfate; CisPt: Cisplatin; CPT: Camptotechin; CYT: Cytarabine; pDHA: Polycarbonate of dihydroxyacetone; pDNA: Plasmid DNA; DOX: Doxorubicin; DTX: Docetaxel; GEM: Gemcitabine; GM-CSF: Granulocyte-macrophage colony-stimulating factor; HA: Hyaluronic acid; Hep: Heparin; LAO: Lactic acid oligomers; Lys: Lysine; ME: 2-Methoxyestradiol; MMT: Montmorillonite; bis-MPA: 2,2-bis(methylol)propionic acid; MPEG: Monomethoxy poly(ethylene glycol); MPL: Monophosphoryl lipid A; MTX: Methotrexate; OVA: Ovalbumin; OXA: Oxaliplatin; PAA: Poly(acrylic acid); PAEU: Poly(β-aminoester urethane); PCB: Polycarbonate; PCL: Poly(*ε*-caprolactone); PCLA: Poly(*ε*-caprolactone-*co*-lactide); PCLMA: Poly(e-caprolactone) monomethacrylate; PEG: Polyethylene glycol; PEGMA: Methoxypoly(ethylene glycol) monomethacrylate; PEI: Poly(ethylene imine); PL: Pluronic L; PLA: Polylactide; PLGA: Poly(lactide-*co*-glycolide); POA: Poly(octadecanedioic anhydride); PPFEMA: Poly(2-(perfluorobutyl)ethyl methacrylate); PTX: Paclitaxel; PUSSM: Poly(urethane sulfide-sulfamethazine); QA: Quil A; SLN: Solid lipid nanoparticles; SMA: Sulfamethazine-acrylamide; TPEGT: Thymine-terminated poly(ethylene glycol); TPT: Topotecan; TZB: Trastuzumab; Vit: Vitamin.

**Table 4:** Composition and anticancer performance of injectable hydrogels based on polyacrylates.

Ref	Composition		Carrier Properties		Delivery Properties		Cancer Model			Health Model	
	Hydrogel (Gelation Process)	Composite Component	Degradation Time (Days)	Smart Responsivity	Bioactive Agent (DL% *w/w*)	Release Time (Days)	Type	In Vitro	In Vivo	In Vitro	In Vivo
[132]	PEGDMA (Chemical – RP)	PEG–PCL	- - -	- - -	TMZ* (0.9–1.3)	12	Glioblastoma	- - -	U87	- - -	- - -
[133]	PEGDMA (Chemical – RP)	PLGA	- - -	- - -	PTX* (4.0)	7	Glioblastoma	U87	U87	- - -	Mice
[134]	PEGDMA (Chemical – RP)	TiO_2_MWCNT	- - -	NIR	DOX* (10)	3	Breast	MCF7	MCF7	- - -	- - -
[135]	PEGDMA (Physical – T)	ZnFe_2_O_4_	- - -	Magnetic	- - -	- - -	Breast	- - -	4T1	- - -	- - -
[136]	p(MEO_2_MA-OEGMA-AA) (Chemical – RP)	- - -	- - -	- - -	BSA (1.6) Epo (1.6)	3 1.5	Prostate	- - -	PC3	3T3	- - -
[137]	PMNT–PEG–PMNT/PAA (Physical)	- - -	- - -	- - -	IL-12 (0.07)	15	Murine Colon	- - -	C26	- - -	- - -
[138]	p(NIPAAm-MPCD) (Chemical – RP)	AuNRs	- - -	pH NIR	DOX (6.6)	30	Breast	MCF7	- - -	- - -	- - -
							Cervix	HeLa	- - -		
							Murine sarcoma	- - -	S180		
[139]	PNAm (Chemical – RP)	PDA–AuRNs	- - -	NIR	DOX	2^§^	Murine breast	- - -	4T1	- - -	- - -

* Loaded into Composite Component; DL: Drug loading; RP: Radical polymerization; AuNRs: Gold nanorods; BSA: Bovine serum albumin; DOX: Doxorubicin; Epo: Erythropoietin; IL: Interleukin; MEO_2_MA: Methyl ether methacrylate; MPCD: Methacrylated poly-*β*-cyclodextrin; MWCNT: Multi-walled carbon nanotubes; NIPAAm: N-isopropyl acrylamide; NIR: Near-infrared; OEGMA: Poly(ethylene glycol) methyl ether methacrylate; PAA: Poly(acrylic acid); PCL: Poly(*ε*-caprolactone); PDA: Polydopamine; PEG: Polyethylene glycol; PEGDMA: Polyethylen glycole dimethacrylate; PLGA: Poly(lactide-*co*-glycolide); PMNT: Poly[4-(2,2,6,6-tetramethyl piperidine-*N*-oxyl)aminomethylstyrene; PNAm: Poly(*N*-acryloylglycinamide-*co*-acrylamide); PTX: Paclitaxel; TMZ: Temozolomide.

**Table 5:** Composition and anticancer performance of injectable hydrogels based on synthetic polypeptide.

Ref	Composition		Carrier Properties		Delivery Properties		Cancer Model			Health Model	
	Hydrogel (Gelation Process)	Composite Component	Degradation Time (Days)	Smart Responsivity	Bioactive Agent (DL% *w/w*)	Release Time (Days)	Type	In Vitro	In Vivo	In Vitro	In Vivo
[146]	Fmoc-FF/PLL (Physical – I)	- - -	- - -	- - -	Ce6 (0.4)	14#	Breast	- - -	MCF7	- - -	- - -
[147]	Fmoc-FF/PLL (Physical – I)	- - -	- - -	- - -	- - -	- - -	- - -	- - -	- - -	- - -	Mice
[148]	RGD–PIC (Physical – T)	- - -	- - -	- - -	- - -	- - -	- - -	- - -	- - -	- - -	Mice
[149]	AcVES3 (Physical – T)	- - -	- - -	- - -	TIMP-2 (4.0)	28	Lung	A549	- - -	- - -	- - -
[150]	FEFFFK (Physical – T)	- - -	- - -	- - -	DOX (0.5)	20#	Breast	MDA-MB 231	MDA-MB 231	- - -	- - -
							Murine breast	4T1	4T1		
[151]	Nap-GFFYGRGDH_n_ (n = 0–2) (Physical – T)	- - -	- - -	pH	DOX** (3.9–12.4)	7	Lung	A549	- - -	- - -	Mice
							Cervix	HeLa			
							Breast	MCF-7			
[152]	K_2_(SL)_6_K_2_ (Physical – T)	- - -	- - -	- - -	CDN (40)	1	Murine Oral	MOC2-E6E7	MOC2-E6E7	- - -	- - -
[153]	(RADA)_8_ (Physical – T)	- - -	10	- - -	MEL** DOX (16)	7	Murine Melanoma	B16F10	B16F10	- - -	- - -
[154]	C_16_-GNNQQNYKD-OH (Physical – T)	Liposome	- - -	- - -	LST DOX*	9	Breast	- - -	4T1	- - -	- - -
[155]	PEG–PAH (Physical – T)	- - -	- - -	pH	DOX (1.7)	2	Fibrosarcoma	HT1080	HT1080	3T3	- - -
[156]	MPEG–(PELG–LG) (Physical – T)	- - -	28	- - -	CisPt (1.0)	7	Murine colon	C26	C26	- - -	- - -
							Cervix	HeLa	- - -		
							Breast	MCF-7	- - -		
[157]	MPEG–PV (Physical – T)	- - -	- - -	- - -	TCL (50)	21#	Murine Melanoma	- - -	B16	- - -	- - -
[158]	MPEG–PAF (Physical – T)	- - -	28	Temperature	DOX (6.0) CA4 (6.0)	28	Murine cervix	- - -	U14	- - -	- - -
[163]	MPEG–PLD–Arg-α–CD (Physical)	- - -	- - -	- - -	sRNA	6	Epithelium	HNE-1	HNE-1	3T3	- - -
[159]	PELG–PEG–PELG (Physical – T)	- - -	14	- - -	PTX (6.0)	21	Liver	HEPG2	HEPG2	- - -	Mice
[15]	(Me-D-1MT)–PEG–(Me-D-1MT) (Physical – T)	- - -	21	ROS	PD-1/PD-L1 CTLA-4	16#	Murine Melanoma	B16F10	B16F10	- - -	- - -
[160]	FFE–EE (Physical – Red)	- - -	- - -	Redox	SN-38**	3	Breast	MD-MBA-231	- - -	- - -	- - -
[161]	KE–EE/AcKE–EE/E–EE/R–EE/S–EE (Physical – Red)	- - -	- - -	Redox	DXM** TX**	1	Liver	HepG2	- - -	- - -	- - -

* Loaded to composite component; ** conjugated to hydrogel; # from in vivo experiments; DL: Drug loading; I: Ionic; T: Temperature; Red: Redox; Ac: Acetyl; AcVES3: Ac−VEVSVSVEV^D^PPTEVSVEVEV−NH_2_; Arg: Arginine; C_16_: Palmitic acid; CA4: Combretastatin CDN: Cyclic dinucleotides; CisPt: Cisplatin; CTLA-4: Cytotoxic T lymphocyte antigen 4; D-1MT: Dextro-1methyl tryptophan; DOX: Doxorubicin; DXM: Dexamethasone; Fmoc-FF: *N*-fluorenylmethoxycarbonyl diphenylalanine; LG: L-glutamic acid; LST: Losartan; MEL: Melittin; MPEG: Monomethoxy poly(ethylene glycol); Nap: Naphthylacetic acid; PAF: Poly(alanine-phenylalanine); PAH: α,β-polyaspartyl hydrazide; PD-1/PD-L1: Programmed cell death protein 1/programmed cell death-ligand 1; PEG: Polyethylene glycol; PELG: Poly(ethyl-L-glutamate); PIC: Tri-ethylene glycol-substituted polyisocyanopeptide; PLL: Poly-*L*-lysine; PTX: Paclitaxel; PV: Poly(*L*-valine); ROS: Reactive oxygen species; SN-38: 7-ethyl-10-hydroxycamptothecin; TIMP-2: Tissue inhibitor of metalloproteinase 2; TLC: Tumor cell lysates; TX: Taxol.

**Table 6:** Composition and anticancer performance of injectable hydrogels based on dendrimers and other systems.

Ref	Composition		Carrier Properties		Delivery Properties		Cancer Model			Health Model	
	Hydrogel (Gelation Process)	Composite Component	Degradation Time (Days)	Smart Responsivity	Bioactive Agent (DL% *w/w*)	Release Time (Days)	Type	In Vitro	In Vivo	In Vitro	In Vivo
**Dendrimers**											
[170]	PGA–MA/PEG-4–SH (Chemical – MR)	- - -	- - -	- - -	TZB (1.0–13)	42	Breast	BT474	BT474	- - -	- - -
[171]	HPAE (Chemical – RP)	- - -	7	- - -	DOX (0.05)	5	Breast	MCF7	- - -	L929	- - -
					5-FU (0.5)						
					LC (0.2)						
[172]	PAMAM(G4)/PEG–PDBCO (Physical)	- - -	- - -	- - -	5-FU (4)	0.5	Head/Neck	HN12	HN12	3T3	- - -
[173]	PEGDA–PAMAM (Chemical – MR)	- - -	14-22 1.4-2.0	pH Redox	DOX (4)	2	Cervix	HeLa	- - -	- - -	- - -
[174]	PEG-4–SH/PDMA–PEGMA (Physical)	p(AA-co-4-VPBA)	21	pH Redox	CA4P (0.05) DOX* (14.3–96.3)	3 14	Breast	MCF-7	- - -	3T3	- - -
							Liver	- - -	HepG2		
[175]	PEG-4–SH/PEG-2–MI (Physical – pH)	- - -	2.7	Redox	BSA (1.2)	7	- - -	- - -	- - -	- - -	- - -
[176]	PEGBA-4/PEGBA-8 (Chemical – RP)	PEGBA-4/PEGBA-8	- - -	pH	PLP*	20	Oral	CAL-27	- - -	- - -	- - -
[177]	PBA–PEG (Physical)	- - -	- - -	pH	DOX (1.2)	5	Murine Breast	4T1	4T1	3T3	- - -
[34]	PEG-4–SH/PLL (Chemical – C)	- - -	14	pH	MTF (5.0) 5-FU (0.5)	14	Colon	C26	C26	- - -	- - -
[178]	Hep–PEG-4–SH (Physical)	- - -	- - -	- - -	DOX (0.004–0.08)	4	Breast	MCF-7	MDA-MB-231	- - -	- - -
								MDA-MB-231			
**Other systems**											
[181]	PVA/GO (Physical)	β-CD	- - -	pH	CPT (5.0)	5	- - -	- - -	- - -	- - -	- - -
[180]	GemC_12_LNC (Physical – PI)	- - -	- - -	- - -	- - -	- - -	Glioblastoma	U251	- - -	- - -	- - -
								T98-G	- - -		
								9L-LacZ	- - -		
								U-87	U87		

* Loaded into composite component; DL: Drug loading; MR: Michael reaction; RP: Radical polymerization; 4-VPBA: 4-vinylboronic Acid; 5-FU: 5-Fluoruracil; CD: Cyclodextrin; AA: Acrylic acid; BSA: Bovine serum albumin; CA4P: Combrestatin A4 phosphate; DOX: Doxorubicin; GO: Graphene oxide; Hep: Heparin; HPAE: Hyperbranched poly(amine-ester); LC: Leucovorin calcium; LNC: Lipod nanocapsule; MPEG: Monomethoxy poly(ethylene glycol); MTF: Metformin; PAMAM: Polyamidoamine; PBA: Phenylboronic acid; pDMA: Poly(3,4-dihydroxyphenethyl)-methacrylamide; PEG-4-SH: 4-arm PEG; PEGBA-4: 4-arm PEG-boronic acid; PEGBA-8: 8-arm PEG-boronic acid; PEGDA: PEG-based diacrylate; PGA-MA: Maleimide-modified c-polyglutamic acid; PEGMA: Methoxypoly(ethylene glycol) monomethacrylate; PEG-2-MI: Maleimide-functionalized linear PEG; PLD-Arg: Arginine-poly(*L*-lysine) dendron; PLL: Poly-*L*-lysine; PLP: Polyphenols mixture; PVA: Polyvinyl alcohol.

**Table 7:** Composition and anticancer performance of injectable hydrogels based on chitosan.

Ref	Composition		Carrier Properties		Delivery Properties		Cancer Model			Health Model	
	Hydrogel (Gelation Process)	Composite Component	Degradation Time (Days)	Smart Responsivity	Bioactive Agent (DL% *w/w*)	Release Time (Days)	Type	In Vitro	In Vivo	In Vitro	In Vivo
**Naturals**											
[190]	CS (Chemical – C)	TA-ZnPc	1	Light pH	TA-ZnPc (6.0)	8	Breast	MDA-MB-231	- - -	- - -	- - -
							Melanoma	A435			
[191]	CS/β-GP (Physical – T)	- - -	- - -	- - -	CisPt (1.0)	15	Colon	HCT-116	- - -	- - -	- - -
							Breast	MCF7			
[193]	CS/β-GP/HA (Physical – I)	- - -	- - -	pH	DOX (0.016–0.033)	5	Cervix	HeLa	- - -	- - -	- - -
[194]	CS/β-GP/CNT (Physical – T)	- - -	21	- - -	MTX	7	Breast	MCF7	- - -	3T3	- - -
[196]	CS/β-GP (Physical – T)	MPEG	- - -	- - -	MEL** (4.6–10.6)	4	- - -	- - -	- - -	- - -	- - -
[198]	CS/β-GP (Physical – T)	Liposome	- - -	pH	TPT* (0.97)	2	Murine Liver	- - -	H22	- - -	- - -
[197]	CS/β-GP (Physical – T)	Liposome	- - -	- - -	DOX* (4.5)	7	Ovarian	A2780	- - -	- - -	- - -
[195]	CS/β-GP (Physical – T)	Liposome	- - -	- - -	DOX* (0.2) ^188^Re*	21	Murine Breast	- - -	4T1	- - -	- - -
[199]	CS/β-GP (Physical – T)	Sn	- - -	- - -	DOX (0.025) ^188^Re	2	Liver	N1-S1	N1-S1	- - -	- - -
[200]	CS/β-GP (Physical – T)	Fe_3_O_4_	48	Magnetic	BCG	- - -	Bladder	- - -	Mice	- - -	- - -
[201]	CS/β-GP (Physical – T)	F_3_O_4_	- - -	Magnetic	- - -	- - -	Breast	- - -	SK-BR-3	- - -	- - -
							Ovarian		SKOV-3		
	ALG (Physical – T)						Glioblastoma		LN229		
							Colon		Co112		
									T380		
[202]	CS/β-GP (Physical – T)	GO/PEI–Fe_3_O_4_	- - -	pH Magnetic	DOX* (200)	0.5	Breast	MCF7	- - -	- - -	- - -
							Murine Sarcoma	- - -	S180		
[203]	CS/G (Physical – T)	- - -	21	- - -	MTX (0.0125)	7.5	Breast	MCF7	- - -	- - -	- - -
[204]	CS-ALG (Physical – I)	- - -	31	- - -	anti-VEGF (0.018)	- - -	- - -	- - -	- - -	HUVECs	- - -
[212]	HBCS (Physical)	- - -	45	- - -	DOX (2.5–10)	3	Murine Breast	4-T1	- - -	HUVEC	- - -
[214]	CMCS-oxALG (Chemical – C)	- - -	- - -	- - -	HDBP	- - -	Liver	Bel-7402	- - -	L02	- - -
							Murine Liver	H-22	H-22		
[216]	CECS-oxALG (Chemical – C)	MGM	14	Magnetic	5-FU* (4.0)	35	- - -	- - -	- - -	- - -	- - -
[217]	CECS/HA (Chemical – C)	- - -	10	pH	DOX (0.3)	3.5	Cervix	HeLa	- - -	- - -	- - -
[218]	CS–oxDEX (Chemical – C)	PF127	10	pH Redox	5-FU (2.5) CUR* (7.6)	10	Cervix	HeLa	- - -	- - -	- - -
[219]	PBCS–oxDEX (Physical – T)	- - -	- - -	pH Glucose	DOX (1.0)	0.5	- - -	- - -	- - -	L929	- - -
[220]	CS–DA/oxPLN (Chemical – C)	- - -	- - -	pH	DOX (0.01–0.32) AMX (0.5)	2.5 1.5	Colon	HCT116	- - -	- - -	- - -
[221]	SCS–oxCS (Chemical – C)	- - -	11	pH	DOX (3.0) FeG1 (5.0)	6 2	- - -	- - -	- - -	MSC	- - -
[222]	SCS–oxALG (Physical)	- - -	- - -	pH	DOX** (7.6)	2	Breast	MCF7	- - -	- - -	- - -
									MDA-MB-231		
[223]	GTMACS/ePC/LA (Physical)	- - -	- - -	- - -	DTX	- - -	- - -	- - -	- - -	- - -	mice
[224]	CS–CAT (Physical – I)	- - -	- - -	- - -	DOX DTX (2.5)	18	Murine Lung	- - -	LLC	C212	- - -
							Murine Breast	4T1	4T1		
[225]	CS–TRIPOD (Chemical – C)	- - -	- - -	Light pH	TPP**	12	Breast	MCF7	- - -	- - -	- - -
							Liver	HepG2			
**N/S Hybrids**											
[192]	CS/β-GP/NIPAAm–IA (Physical – T)			pH Thermo	DOX (3.0)	8	Breast	MCF7	- - -	- - -	- - -
[205]	CS–HA–NIPAAm (Chemical – C)	- - -	40	pH	DOX (10)	12	Murine Colon	CT-26	CT-26	- - -	- - -
[206]	CS–HA–NIPAAm (Physical – T)	GO	60	pH	DOX* (14.20)	9	Breast	MCF7	MCF7	- - -	- - -
[207]	GCS/GMA (Chemical – IRD)	- - -	- - -	- - -	DOX (1.0)	7	Breast	MCF7	MCF7	- - -	- - -
[208]	GCS–PEG (Physical – T)	- - -	- - -	- - -	CRB (2.0)	0.25	- - -	- - -	- - -	- - -	mice
[209]	GCS–PEG (Physical – T)	- - -	- - -	- - -	TMPyP (0.05–0.2)	7#	Cervix	U14	U14	- - -	- - -
[210]	GCS–PEG (Chemical – C)	PLGA-F_3_O_4_	- - -	Magnetic	DTX* (9.0)	30	Breast	MDA-MB-231	MDA-MB-231	- - -	Mice
[211]	GCS/PF127/α-CD (Physical)	- - -	11	pH	DOX (1.0–5.0)	8	Liver	HepG2	- - -	- - -	- - -
							Murine Liver	- - -	H22		
[7]	PPLG–HPCS–PPLL (Physical – I)	oxDEX	21	- - -	DOX* (22.1) IL-2* (8.3) IFN-γ* (8.7)	24#	Breast	MCF7	- - -	- - -	- - -
							Cervix	HeLa			
							Lung	A549	A549		
[213]	CMCS–NIPAAm (Chemical – RP)	- - -	- - -	pH Thermo	5-FU (6.2–8.9)	2	Breast	MCF-7	- - -	L929	- - -
							Cervix	HeLa			
[215]	CECS–PEG (Chemical – C)	- - -	8	pH	DOX	7.5	Liver	HepG2	- - -	L929	- - -
[226]	TCS–PEGDMA (Chemical – MR)	STC	- - -	Enzyme	CUR (3.8) LSZ*	7 0.5	Liver	HepG2	HepG2	- - -	- - -

* Loaded in composite component; ** conjugated to composite component; # from in vivo experiments; C: Condensation; I: Ionic; IRD: Irradiation; MR: Michael reaction; RP: Radical polymerizarion; T: Temperature; 5-FU: 5-Fluoruracil; CD: Cyclodextrin; β-GP: β-Glycerophosphate; ALG: Alginate; AMX: Amoxicillin; BCG: Bacillus Calmette–Guérin; CAT: Catechol; CECS: Carboxyethyl chitosan; CisPt: Cisplatin; CMCS: Carboxymethyl chitosan; CNT: Carbon nanotubes; CRB: Carbazochrome; CS: Chitosan; CUR: Curcumin; DA: Dihydrocaffeic acid; DOX: Doxorubicin; DTX: Docetaxel; ePC: Egg phosphatidylcholine; FeG1: Non-hormonal contraceptive; G: Graphene; GCS: Glycol chitosan; GMA: Glycidyl methacrylate; GO: Graphene oxide; GTMACS: Glycidyltrimethylammonium chitosan; HA: Hyaluronic acid; HBCS: Hydroxybutyl chitosan; HDBP: Hydrogel degradation by-product; HPCS: Hydroxypropyl chitosan; IA: Itaconic acid; IL: Interleukin; IFN: Interferon; LA: Lauric aldehyde; LSZ: Lysozyme; MEL: Melphalan; MGM: Magnetic gelatin microspheres; MPEG: Monomethoxy poly(ethylene glycol); MTX: Methotrexate; NIPAAm: *N*-isopropyl acrylamide; oxALG: Oxidized alginate; oxCS: Oxidized chitosan; oxDEX: Oxidized dextran; oxPLN: Oxidized pullulan; PBCS: Phenylboronic-modified chitosan; PEG: Polyethylene glycol; PEGDMA: Polyethilenen glycol dimethacrylate; PEI: Poly(ethylene imine); PF: Pluronic F; PLGA: Poly(lactide-*co-*glycolide); PPLG: 4-Arm PEG-polyglutammic acid; PPLL: 4-arm poly(ethylene glycol)-poly(*L*-lysine); SCS: Succinate chitosan; STC: Starch; TA-ZnPc: Tetra-aldehyde functionalized zinc phthalocyanine; TCS: Thiolated chitosan; TMPyP: Meso-tetrakis(1-methylpyridinium-4-yl) porphyrin; PP: Tetrakis(4-aminophenyl)porphyrin; TPT: Topotecan; TRIPOD: 2,4,6-tris(p-formylphenoxy)-1,3,5-triazine; VEGF: Vascular endothelial growth factor

**Table 8:** Composition and anticancer performance of injectable hydrogels based on hyaluronic acid.

Ref	Composition		Carrier Properties		Delivery Properties		Cancer Model			Health Model	
	Hydrogel (Gelation Process)	Composite Component	Degradation Time (Days)	Smart Responsivity	Bioactive Agent (DL% *w/w*)	Release Time (Days)	Type	In Vitro	In Vivo	In Vitro	In Vivo
**Naturals**											
[232]	oxHA (Chemical – C)	- - -	10	- - -	Anti-2B11	24#	Breast	MCF7	- - -	- - -	- - -
								MDA-MB-231	MDA-MB-231		
							Murine Breast	- - -	BT-474		
							Murine Melanoma	- - -	B16		
[233]	oxHA (Chemical – C)	HA-IDA HA-MA	- - -	- - -	CisPt* (200)	7.5	Stomach	- - -	MKN45P	- - -	- - -
[236]	HA–Tyr (Chemical – HRP)	- - -		- - -	IFN-α	1	Kidney	ACHN	ACHN	- - -	- - -
[234]	HA–Tyr (Chemical – HRP)	- - -	- - -	Enzyme (HAse)	IFN-α	1	Liver	HAK-1B	HAK-1B	- - -	- - -
[235]	HA–Tyr (Chemical – HRP)	- - -	28	Enzyme (HAse)	TZB (0.3)	28	Breast	BT474	BT474	- - -	- - -
[237]	HA–SH (Chemical – Red)	- - -	- - -	Redox	DOX (1.0)	21	Breast	MCF7	- - -	- - -	- - -
					DOX/SRB (1.0/1.0)			MDA-MB-231			
					DOX/SRB/MTF (1.0/1.0/1.0)		Murine Breast	4T1	4T1		
[238]	HA (Physical – pH)	MSNs	- - -	Enzyme (HAse)	DOX* (27)	6	Breast	SKBR3	- - -	293T	- - -
[239]	HA–αCD (Physical)	AuBNs-MSNs	7	Enzyme (HAse)	DOX* (11.1–32.0)	7	Squamous Carcinoma	SCC	- - -	HaCaT	- - -
**N/S Hybrids**											
[231]	HA/PF127 (Physical – T)	- - -	31	- - -	DOX (0.5)	31	Breast	MCF7	- - -	- - -	mice
[68]	PF127/HA (Physical – T)	- - -	- - -	- - -	DOX (1.0)	1	Murine colon	C26	C26	- - -	- - -
							Colon	HT29	- - -		
							Breast	MCF7	- - -		
[67]	PF127/HA (Physical – T)	PF127_PL121	- - -	- - -	DOX (1.0) DTX* (1.6)	3	Colorectal	- - -	CT26	- - -	- - -
[240]	HA–Gln/PEG-8–SH–Lys (Chemical – E)	- - -	- - -	- - -	- - -	- - -	Breast	MCF7	- - -	C2C12	- - -

* Loaded in composite component; # from in vivo experiments; C: Condensation; E: Enzymatic; Red: Redox; T: Temperature; CD: Cyclodextrin; AuBNs: Gold nanobipyramids; CisPt: Cisplatin; DOX: Doxorubicin; DTX: Docetaxel; Gln: Glutamine substrate peptide; HA: Hyaluronic acid; HAase: Hyaluronidase; HA-IDA: HA-iminodiacetic Acid; HA-MA: HA-malonic acid; HRP: Horseradish peroxidase; IFN: Interferon; Lys: Lysine; MSNs: Mesoporous silica nanoparticles; MTF: Metformin; oxHA: Oxidized HA; PEG-8-SH: 8-arm PEG; PF: Pluronic F; PL: PLuronic L; SRB: Sorafenib; Tyr: Tyramine; TZB: Trastuzumab.

**Table 9:** Composition and anticancer performance of injectable hydrogels based on other polysaccharides.

Ref	Composition		Carrier Properties		Delivery Properties		Cancer Model			Health Model	
	Hydrogel (Gelation Process)	Composite Component	Degradation Time (Days)	Smart Responsivity	Bioactive Agent (DL% *w/w*)	Release Time (Days)	Type	In Vitro	In Vivo	In Vitro	In Vivo
**Naturals**											
[243]	QCL–CCNCs (Physical – I)	- - -	18	- - -	DOX (0.5)	21	Murine Liver	- - -	H22	COS-7	- - -
[245]	CL (Chemical – C)	BPNs	- - -	- - -	- - -	- - -	Murine Melanoma	B16	- - -	J774A.1	- - -
							Liver	SMMC-7721	SMMC-7721		
[248]	ALG (Physical – I)	PAMAM	- - -	- - -	CisPt* (37.0)	30#	Breast	MFC7	- - -	3T3	- - -
								MDA-MB-231			
							Lung	PC9	PC9		
[253]	oxDEX–SRC (Chemical – C)	- - -	70	- - -	HRP (0.39–1.36) DOX (0.39–1.36)	50 30	Melanoma	- - -	B16F10	C2C12	mice
										HL7702	
[254]	oxDEX (Chemical – C)	PAMAM	14	- - -	Pt*	9#	Breast	MDA-MB-231	MDA-MB-231	- - -	mice
[255]	MADEX–SH/MAHA (Chemical – Red)	Bi NPs	- - -	- - -	DOX* (3.1)	7.5	Murine Breast	4T1	4T1	- - -	- - -
[256]	GG (Physical)	Liposome	- - -	- - -	PTX (33)	2	Bladder	T24	- - -	- - -	mice
							Murine Bladder	NBT-II			
[257]	GG (Physical)	CuS NPs	- - -	NIR	DOX* (0.1)	0.2	Murine Breast	4T1	4T1	- - -	- - -
[258]	AGR (Physical)	- - -	7	pH NIR	DOX (4.5)	2	Murine Breast	4T1	4T1	L929	- - -
							Cervix	HeLa	- - -	HUVEC	
[259]	AGR (Physical)	DEX-SH	- - -	- - -	DOX* (20–50)	80	Breast	MDA-MB-231	- - -	3T3	- - -
**N/S Hybrids**											
[244]	HPMCL/PF127/ALG (Physical)	MPEG–DPPE	- - -	- - -	PTX* (5.1) TMZ* (5.3)	3	Murine Glioma	C6	C6	- - -	- - -
[249]	ALG–NIPAAm (Physical – T)	- - -	365	Thermo	DNA	29	Prostate	PC3	- - -	- - -	- - -
[250]	ALG–NIPAAm (Physical – T)	- - -	- - -	Thermo	DOX (1.2)	21	Prostate	AT3B-1N	- - -	- - -	- - -
								AT3B-1			
[251]	oxALG–PEI (Physical)	PLGA–PLA	- - -	- - -	CisPt* (0.01–2.48) PTX* (1.0–1.7)	45	Breast	MDA-MB-231	- - -	- - -	- - -
[252]	oxALG–PEI (Physical)	PLGA–PLA	- - -	- - -	CisPt* (0.01–2.48) PTX* (1.49–1.70)	45	Liver	HepG2	- - -	MRC-5	- - -
[260]	DEX–HEMA/PEI–MA (Chemical – RP)	- - -	9–17	- - -	sRNA	9–17	- - -	- - -	- - -	HEK 293	- - -

* Loaded in composite component; # from in vivo experiments; C: Condensation; I: Ionic; T: Temperature; Red: Redox; RP: Radical polymerization; AGR: Agarose; ALG: Alginate; BPNs: Black phosphorus nanosheets; CCNCs: Cationic cellulose nanocrystals; CisPt: Cisplatin; CL: Cellulose; DEX: Dextran; DOX: Doxorubicin; DPPE: Dipalmitoylphosphatidyle-thanoiamine; GG: Gellan gum; HPMCL: Hydroxypropyl methyl cellulose; HRP: Horseradish peroxidase; MADEX: Methacrylated DEX; MAHA: Methacrylated HA; MPEG: Monomethoxy poly(ethylene glycol); NIPAAm: *N*-isopropyl acrylamide; NIR: Near-infrared; NPs: Nanoparticles; oxALG: Oxidized alginate; oxDEX: Oxidized dextran; PAMAM: Polyamidoamine dendrimer; PEI: Poly(ethylene imine); PF: Pluronic F; PLA: Polylactide; PLGA: Poly(lactide-*co*-glycolide); PTX: Paclitaxel; QCL: Quaternized cellulose; SRC: Sericin; TMZ: Temozolomide.

**Table 10:** Composition and anticancer performance of injectable hydrogels based on proteins.

Ref	Composition		Carrier Properties		Delivery Properties		Cancer Model			Health Model	
	Hydrogel (Gelation Process)	Composite Component	Degradation Time (Days)	Smart Responsivity	Bioactive Agent (DL% *w/w*)	Release Time (Days)	Type	In Vitro	In Vivo	In Vitro	In Vivo
**Naturals**											
[269]	BSA (Chemical – CR)	- - -	3	- - -	DOX (0.11–0.14)	5	Cervix	HeLa	- - -	- - -	- - -
							Breast	MCF-7			
								MDA-MB 231			
[277]	GEL (Chemical – E)	- - -	- - -	- - -	AraC	- - -	Prostate	DU-145	- - -	L929	mice
[274]	GEL–HPA (Chemical – HRP)	- - -	- - -	Enzyme (COLase)	DCs Ad	19#	Murine Lung	- - -	LL	- - -	- - -
[275]	GEL–SBP (Chemical – HRP)	- - -	- - -	- - -	DOX (0.9)	6	Murine Melanoma	- - -	B16F10	- - -	- - -
[276]	GEL–CS (Chemical - C)	Liposome	- - -	- - -	CAL (0.39–0.47) CAL*	7 21	- - -	- - -	- - -	- - -	- - -
[282]	GEL (Physical – T)	PDA	7	pH Enzyme NIR	DOX*	7	Murine Breast	- - -	4T1	- - -	- - -
[286]	SF (Physical – US)	SF NPs	- - -	- - -	SAL* (12) PTX* (12)	5 28	Murine Liver	- - -	H22	- - -	mice
[287]	SF (Physical – CDP)	- - -	- - -	pH	DOX (8–24)	56	Breast	MDA-MB-231	MDA-MB-231	- - -	- - -
[288]	SF (Physical – T)	GO	- - -	- - -	NaLuF_4_:Er^3+^,Yb^3+^	- - -	Murine Breast	4T1	4T1	- - -	- - -
[290]	SELP (Physical – T)	- - -	- - -	- - -	^131^I	- - -	Prostate	- - -	PC3	- - -	- - -
							Pancreas		BxPc3		
[291]	SELP (Physical – T)	- - -	- - -	- - -	DOX (21–28) SRB (21–28)	15-30	- - -	- - -	- - -	- - -	- - -
[292]	SELP (Physical – T)	- - -	- - -	- - -	HSVtk/GCV	- - -	- - -	- - -	- - -	- - -	mice
**N/S Hybrids**											
[270]	HSA–SH/PEG-4–SH (Physical – T)	- - -	21	- - -	TRAIL (5.8)	7	Pancreas	Mia PAca-2	Mia PAca-2	- - -	- - -
[271]	PEG–BSA (Physical – T)	PRNP	50	- - -	PTX* (22.1)	6	Stomach	MKN45	MKN-45	- - -	- - -
[279]	GEL–SWCNT–pNIPAAm (Chemical – RP)	- - -	- - -	Temperature	DOX (1.11)	28	Stomach	BGC-823	BGC-823	- - -	- - -

* Loaded in composite component; # from in vivo experiments; C: Condensation; CDP: Concentration dilution process; CR: Cross-linking; E: Enzyme; US: Ultrasound; RP: Radical polymerization; T: Temperature; Ad: Oncolytic adenovirus; AraC: Cytosine arabinoside; BSA: Bovine serum albumin; CAL: Calcein; COLase: Collagenase; CS: Chitosan; DCs: Dendritic cells; DOX: Doxorubicin; GCV: Ganciclovir; GEL: Gelatin; HPA: Hydroxyphenyl propionic acid; GO: Graphene oxide; HPA: Hydroxyphenyl propionic acid; HRP: Horseradish peroxidase; HSA: Human serum albumin; HSVtk: Herpes simplex virus thymidine kinase; PDA: Polydopamine; PEG-4-SH: 4-arm PEG; pNIPAAm: Poly(N-isopropyl acrylamide); PRNP: Red blood cell membrane nanoparticles; PTX: Paclitaxel; SAL: Salinomycin; SBP: Sugar beet pectin; SELP: Silk–elastin-like protein; SF: Silk fibroin; SRB: Sorafenib; SWCNT: Single-walled carbon nanotubes; TRAIL: Tumor necrosis factor-related apoptosis-inducing ligand.

## References

[1] Li Y., Rodrigues J., Tomás H. (2012). Injectable and biodegradable hydrogels: Gelation, biodegradation and biomedical applications. Chem. Soc. Rev..

[2] Norouzi M., Nazari B., Miller D.W. (2016). Injectable hydrogel-based drug delivery systems for local cancer therapy. Drug Discov. Today.

[3] Mathew A.P., Uthaman S., Cho K.H., Cho C.S., Park I.K. (2018). Injectable hydrogels for delivering biotherapeutic molecules. Int. J. Biol. Macromol..

[4] Yu S., He C., Chen X. (2018). Injectable Hydrogels as Unique Platforms for Local Chemotherapeutics-Based Combination Antitumor Therapy. Macromol. Biosci..

[5] Ko D.Y., Shinde U.P., Yeon B., Jeong B. (2013). Recent progress of in situ formed gels for biomedical applications. Prog. Polym. Sci..

[6] Qi C., Liu J., Jin Y., Xu L., Wang G., Wang Z., Wang L. (2018). Photo-crosslinkable, injectable sericin hydrogel as 3D biomimetic extracellular matrix for minimally invasive repairing cartilage. Biomaterials.

[7] Wu X., He C., Wu Y., Chen X., Cheng J. (2015). Nanogel-Incorporated Physical and Chemical Hybrid Gels for Highly Effective Chemo-Protein Combination Therapy. Adv. Funct. Mater..

[8] Zhang Z., He C.L., Xu Q.H., Zhuang X.L., Chen X.S. (2018). Preparation of Poly(L-glutamic acid)-based Hydrogels via Diels-Alder Reaction and Study on Their Biomolecule-responsive Properties. Acta. Polym. Sin..

[9] Xu Q., Guo L., Sigen A., Gao Y., Zhou D., Greiser U., Creagh-Flynn J., Zhang H., Dong Y., Cutlar L. (2018). Injectable hyperbranched poly(β-amino ester) hydrogels with on-demand degradation profiles to match wound healing processes. Chem. Sci..

[10] Castro V., Rodríguez H., Albericio F. (2016). CuAAC: An Efficient Click Chemistry Reaction on Solid Phase. ACS Comb. Sci..

[11] Dey P., Hemmati-Sadeghi S., Haag R. (2016). Hydrolytically degradable, dendritic polyglycerol sulfate based injectable hydrogels using strain promoted azide-alkyne cycloaddition reaction. Polym. Chem..

[12] Yang Z., Gao D., Cao Z., Zhang C., Cheng D., Liu J., Shuai X. (2015). Drug and gene co-delivery systems for cancer treatment. Biomater. Sci..

[13] Nezhad-Mokhtari P., Ghorbani M., Roshangar L., Soleimani Rad J. (2019). Chemical gelling of hydrogels-based biological macromolecules for tissue engineering: Photo- and enzymatic-crosslinking methods. Int. J. Biol. Macromol..

[14] Xu Q., He C., Zhang Z., Ren K., Chen X. (2016). Injectable, Biomolecule-Responsive Polypeptide Hydrogels for Cell Encapsulation and Facile Cell Recovery through Triggered Degradation. ACS Appl. Mater. Interfaces.

[15] Yu S., Wang C., Yu J., Wang J., Lu Y., Zhang Y., Zhang X., Hu Q., Sun W., He C. (2018). Injectable Bioresponsive Gel Depot for Enhanced Immune Checkpoint Blockade. Adv. Mater..

[16] Kim S.H., Tan J.P.K., Nederberg F., Fukushima K., Colson J., Yang C., Nelson A., Yang Y.Y., Hedrick J.L. (2010). Hydrogen bonding-enhanced micelle assemblies for drug delivery. Biomaterials.

[17] Huebsch N., Kearney C.J., Zhao X., Kim J., Cezar C.A., Suo Z., Mooney D.J. (2014). Ultrasound-triggered disruption and self-healing of reversibly cross-linked hydrogels for drug delivery and enhanced chemotherapy. Proc. Natl. Acad. Sci. USA.

[18] Appel E.A., Del Barrio J., Loh X.J., Scherman O.A. (2012). Supramolecular polymeric hydrogels. Chem. Soc. Rev..

[19] Bai Y., Li S., Li X., Han X., Li Y., Zhao J., Zhang J., Hou X., Yuan X. (2019). An injectable robust denatured albumin hydrogel formed via double equilibrium reactions. J. Biomater. Sci. Polym. Ed..

[20] Gačanin J., Kovtun A., Fischer S., Schwager V., Quambusch J., Kuan S.L., Liu W., Boldt F., Li C., Yang Z. (2017). Spatiotemporally Controlled Release of Rho-Inhibiting C3 Toxin from a Protein–DNA Hybrid Hydrogel for Targeted Inhibition of Osteoclast Formation and Activity. Adv. Healthc. Mater..

[21] Yan C., Pochan D.J. (2010). Rheological properties of peptide-based hydrogels for biomedical and other applications. Chem. Soc. Rev..

[22] Slaughter B.V., Khurshid S.S., Fisher O.Z., Khademhosseini A., Peppas N.A. (2009). Hydrogels in regenerative medicine. Adv. Mater..

[23] Bakaic E., Smeets N.M.B., Hoare T. (2015). Injectable hydrogels based on poly(ethylene glycol) and derivatives as functional biomaterials. RSC Adv..

[24] Singh N.K., Lee D.S. (2014). In situ gelling pH- and temperature-sensitive biodegradable block copolymer hydrogels for drug delivery. J. Control. Release.

[25] Nguyen M.K., Lee D.S. (2010). Injectable biodegradable hydrogels. Macromol. Biosci..

[26] Tran R.T., Gyawali D., Nair P., Yang J., Sharma S., Mudhoo A. (2011). Biodegradable injectable systems for bone tissue engineering. A Handbook of Applied Biopolymer Technology: Synthesis, Degradation and Applications.

[27] Srinivasan C., Weight A.K., Bussemer T., Klibanov A.M. (2013). Non-aqueous suspensions of antibodies are much less viscous than equally concentrated aqueous solutions. Pharm. Res..

[28] Sun S., Cao H., Su H., Tan T. (2009). Preparation and characterization of a novel injectable in situ cross-linked hydrogel. Polym. Bull..

[29] Tu Y., Chen N., Li C., Liu H., Zhu R., Chen S., Xiao Q., Liu J., Ramakrishna S., He L. (2019). Advances in injectable self-healing biomedical hydrogels. Acta. Biomater..

[30] Kretlow J.D., Klouda L., Mikos A.G. (2007). Injectable matrices and scaffolds for drug delivery in tissue engineering. Adv. Drug Deliv. Rev..

[31] Říhová B. (2000). Immunocompatibility and biocompatibility of cell delivery systems. Adv. Drug Deliv. Rev..

[32] Fu C.X., Lin X.X., Wang J., Zheng X.Q., Li X.Y., Lin Z.F., Lin G.Y. (2016). Injectable micellar supramolecular hydrogel for delivery of hydrophobic anticancer drugs. J. Mater. Sci..

[33] Brigger I., Dubernet C., Couvreur P. (2012). Nanoparticles in cancer therapy and diagnosis. Adv. Drug Deliv. Rev..

[34] Wu X., He C., Wu Y., Chen X. (2016). Synergistic therapeutic effects of Schiff’s base cross-linked injectable hydrogels for local co-delivery of metformin and 5-fluorouracil in a mouse colon carcinoma model. Biomaterials.

[35] Cirillo G., Nicoletta F.P., Curcio M., Spizzirri U.G., Picci N., Iemma F. (2014). Enzyme immobilization on smart polymers: Catalysis on demand. React. Funct. Polym..

[36] Allcock H.R., Morozowich N.L. (2012). Bioerodible polyphosphazenes and their medical potential. Polym. Chem..

[37] Baillargeon A.L., Mequanint K. (2014). Biodegradable polyphosphazene biomaterials for tissue engineering and delivery of therapeutics. BioMed Res. Int..

[38] Hindenlang M.D., Soudakov A.A., Imler G.H., Laurencin C.T., Nair L.S., Allcock H.R. (2010). Iodine-containing radio-opaque polyphosphazenes. Polym. Chem..

[39] Singh A., Krogman N.R., Sethuraman S., Nair L.S., Sturgeon J.L., Brown P.W., Laurencin C.T., Allcock H.R. (2006). Effect of side group chemistry on the properties of biodegradable l-alanine cosubstituted polyphosphazenes. Biomacromolecules.

[40] Cho J.K., Hong K.Y., Park J.W., Yang H.K., Song S.C. (2011). Injectable delivery system of 2-methoxyestradiol for breast cancer therapy using biodegradable thermosensitive poly(organophosphazene) hydrogel. J. Drug Target..

[41] Allcock H.R., Pucher S.R., Scopelianos A.G. (1994). Poly[(amino acid ester)phosphazenes] as substrates for the controlled release of small molecules. Biomaterials.

[42] Teasdale I., Brüggemann O. (2013). Polyphosphazenes: Multifunctional, biodegradable vehicles for drug and gene delivery. Polymers.

[43] Ogueri K.S., Allcock H.R., Laurencin C.T. (2019). Polyphosphazene Polymer. Encycl. Polym. Sci. Technol..

[44] Kwak M.K., Hur K., Yu J.E., Han T.S., Yanagihara K., Kim W.H., Lee S.M., Song S.C., Yang H.K. (2010). Suppression of in vivo tumor growth by using a biodegradable thermosensitive hydrogel polymer containing chemotherapeutic agent. Investig. New Drugs.

[45] Al-Abd A.M., Hong K.Y., Song S.C., Kuh H.J. (2010). Pharmacokinetics of doxorubicin after intratumoral injection using a thermosensitive hydrogel in tumor-bearing mice. J. Control. Release.

[46] Wang J., Wang D., Yan H., Tao L., Wei Y., Li Y., Wang X., Zhao W., Zhang Y., Zhao L. (2017). An injectable ionic hydrogel inducing high temperature hyperthermia for microwave tumor ablation. J. Mater. Chem. B.

[47] Cho J.K., Hong J.M., Han T., Yang H.K., Song S.C. (2013). Injectable and biodegradable poly(organophosphazene) hydrogel as a delivery system of docetaxel for cancer treatment. J. Drug Target..

[48] Kim J.H., Lee J.H., Kim K.S., Na K., Song S.C., Lee J., Kuh H.J. (2013). Intratumoral delivery of paclitaxel using a thermosensitive hydrogel in human tumor xenografts. Arch. Pharmacal Res..

[49] Cho J.K., Kuh H.J., Song S.C. (2014). Injectable poly(organophosphazene) hydrogel system for effective paclitaxel and doxorubicin combination therapy. J. Drug Target..

[50] Kim Y.M., Park M.R., Song S.C. (2013). An injectable cell penetrable nano-polyplex hydrogel for localized siRNA delivery. Biomaterials.

[51] Cho J.K., Chun C., Kuh H.J., Song S.C. (2012). Injectable poly(organophosphazene)-camptothecin conjugate hydrogels: Synthesis, characterization, and antitumor activities. Eur. J. Pharm. Biopharm..

[52] Kim J.I., Kim B., Chun C., Lee S.H., Song S.C. (2012). MRI-monitored long-term therapeutic hydrogel system for brain tumors without surgical resection. Biomaterials.

[53] Zhang Z.Q., Song S.C. (2016). Thermosensitive/superparamagnetic iron oxide nanoparticle-loaded nanocapsule hydrogels for multiple cancer hyperthermia. Biomaterials.

[54] Akash M.S.H., Rehman K. (2015). Recent progress in biomedical applications of pluronic (PF127): Pharmaceutical perspectives. J. Control. Release.

[55] Moebus K., Siepmann J., Bodmeier R. (2009). Alginate-poloxamer microparticles for controlled drug delivery to mucosal tissue. Eur. J. Pharm. Biopharm..

[56] Klouda L. (2015). Thermoresponsive hydrogels in biomedical applications A seven-year update. Eur. J. Pharm. Biopharm..

[57] Cabana A., Aït-Kadi A., Juhász J. (1997). Study of the gelation process of polyethylene oxide(a)-polypropylene oxide(*b*)-polyethylene oxide, copolymer (poloxamer 407) aqueous solutions. J. Colloid Interface Sci..

[58] Thimmaraju M.K., Bheemanapally K., Dharavath R., Kakarla L., Botlagunta M. (2017). Improved anticancer activity of meloxicam hydrogels in K562 and HL60 cell lines. J. Young Pharm..

[59] Hu H., Lin Z., He B., Dai W., Wang X., Wang J., Zhang X., Zhang H., Zhang Q. (2015). A novel localized co-delivery system with lapatinib microparticles and paclitaxel nanoparticles in a peritumorally injectable in situ hydrogel. J. Control. Release.

[60] Xu G., Li B., Wang T., Wan J., Zhang Y., Huang J., Shen Y. (2018). Enhancing the anti-ovarian cancer activity of quercetin using a self-assembling micelle and thermosensitive hydrogel drug delivery system. RSC Adv..

[61] Kim D.Y., Kwon D.Y., Kwon J.S., Park J.H., Park S.H., Oh H.J., Kim J.H., Min B.H., Park K., Kim M.S. (2016). Synergistic anti-tumor activity through combinational intratumoral injection of an in-situ injectable drug depot. Biomaterials.

[62] Zhang N., Xu X., Zhang X., Qu D., Xue L., Mo R., Zhang C. (2016). Nanocomposite hydrogel incorporating gold nanorods and paclitaxel-loaded chitosan micelles for combination photothermal-chemotherapy. Int. J. Pharm..

[63] Fu J.J., Chen M.Y., Li J.X., Zhou J.H., Xie S.N., Yuan P., Tang B., Liu C.C. (2018). Injectable hydrogel encapsulating Cu_2_MnS_2_ nanoplates for photothermal therapy against breast cancer. J. Nanobiotechnology.

[64] Bruschi M.L., Borghi-Pangoni F.B., Junqueira M.V., de Souza Ferreira S.B., Ficai D., Grumezescu A.M., Ficai D., Grumezescu A. (2017). Chapter 12—Nanostructured therapeutic systems with bioadhesive and thermoresponsive properties. Nanostructures for Novel Therapy.

[65] Lin H.R., Tseng C.C., Lin Y.J., Ling M.H. (2012). A novel in-situ-gelling liquid suppository for site-targeting delivery of anti-colorectal cancer drugs. J. Biomater. Sci. Polym. Ed..

[66] Gao L., Wang X., Ma J., Hao D., Wei P., Zhou L., Liu G. (2016). Evaluation of TPGS-modified thermo-sensitive Pluronic PF127 hydrogel as a potential carrier to reverse the resistance of P-gp-overexpressing SMMC-7721 cell lines. Colloids Surf. B Biointerfaces.

[67] Sheu M.T., Jhan H.J., Su C.Y., Chen L.C., Chang C.E., Liu D.Z., Ho H.O. (2016). Codelivery of doxorubicin-containing thermosensitive hydrogels incorporated with docetaxel-loaded mixed micelles enhances local cancer therapy. Colloids Surf. B Biointerfaces.

[68] Jhan H.J., Liu J.J., Chen Y.C., Liu D.Z., Sheu M.T., Ho H.O. (2015). Novel injectable thermosensitive hydrogels for delivering hyaluronic acid-doxorubicin nanocomplexes to locally treat tumors. Nanomedicine.

[69] Khan S., Minhas M.U., Ahmad M., Sohail M. (2018). Self-assembled supramolecular thermoreversible β-cyclodextrin/ethylene glycol injectable hydrogels with difunctional Pluronic^®^127 as controlled delivery depot of curcumin. Development, characterization and in vitro evaluation. J. Biomater. Sci. Polym. Ed..

[70] Hu X., Li D., Tan H., Pan C., Chen X. (2014). Injectable graphene oxide/graphene composite supramolecular hydrogel for delivery of anti-cancer drugs. J. Macromol. Sci. A Pure Appl. Chem..

[71] Moon H.J., Ko D.Y., Park M.H., Joo M.K., Jeong B. (2012). Temperature-responsive compounds as in situ gelling biomedical materials. Chem. Soc. Rev..

[72] Kang Y.M., Kim G.H., Kim J.I., Kim D.Y., Lee B.N., Yoon S.M., Kim J.H., Kim M.S. (2011). In vivo efficacy of an intratumorally injected in situ-forming doxorubicin/poly(ethylene glycol)-b-polycaprolactone diblock copolymer. Biomaterials.

[73] Lei N., Gong C., Qian Z., Luo F., Wang C., Wang H., Wei Y. (2012). Therapeutic application of injectable thermosensitive hydrogel in preventing local breast cancer recurrence and improving incision wound healing in a mouse model. Nanoscale.

[74] Kondiah P.J., Choonara Y.E., Kondiah P.P.D., Marimuthu T., Kumar P., Du Toit L.C., Pillay V. (2016). A review of injectable polymeric hydrogel systems for application in bone tissue engineering. Molecules.

[75] Choi B., Lee M., Nair L.S. (2016). Injectable Hydrogels for Articular Cartilage Regeneration. Injectable Hydrogels for Regenerative Engineering.

[76] Shi K., Wang Y.L., Qu Y., Liao J.F., Chu B.Y., Zhang H.P., Luo F., Qian Z.Y. (2016). Synthesis, characterization, and application of reversible PDLLA-PEG-PDLLA copolymer thermogels in vitro and in vivo. Sci. Rep..

[77] Shi K., Xue B., Jia Y., Yuan L., Han R., Yang F., Peng J., Qian Z. (2019). Sustained co-delivery of gemcitabine and cis-platinum via biodegradable thermo-sensitive hydrogel for synergistic combination therapy of pancreatic cancer. Nano Res..

[78] Fan R., Tong A., Li X., Gao X., Mei L., Zhou L., Zhang X., You C., Guo G. (2015). Enhanced antitumor effects by docetaxel/LL 37-loaded thermosensitive hydrogel nanoparticles in peritoneal carcinomatosis of colorectal cancer. Int. J. Nanomed..

[79] Li X., Fan R., Wang Y., Wu M., Tong A., Shi J., Xiang M., Zhou L., Guo G. (2015). In situ gel-forming dual drug delivery system for synergistic combination therapy of colorectal peritoneal carcinomatosis. RSC Adv..

[80] Liang Y., Dong C., Zhang J., Deng L., Dong A. (2017). A reconstituted thermosensitive hydrogel system based on paclitaxel-loaded amphiphilic copolymer nanoparticles and antitumor efficacy. Drug Dev. Ind. Pharm..

[81] Park M.H., Joo M.K., Choi B.G., Jeong B. (2012). Biodegradable thermogels. Acc. Chem. Res..

[82] Qiu B., Stefanos S., Ma J., Lalloo A., Perry B.A., Leibowitz M.J., Sinko P.J., Stein S. (2003). A hydrogel prepared by in situ cross-linking of a thiol-containing poly(ethylene glycol)-based copolymer: A new biomaterial for protein drug delivery. Biomaterials.

[83] Ma H., He C., Cheng Y., Yang Z., Zang J., Liu J., Chen X. (2015). Localized Co-delivery of Doxorubicin, Cisplatin, and Methotrexate by Thermosensitive Hydrogels for Enhanced Osteosarcoma Treatment. ACS Appl. Mater. Interfaces.

[84] He C., Kim S.W., Lee D.S. (2008). In situ gelling stimuli-sensitive block copolymer hydrogels for drug delivery. J. Control. Release.

[85] Chang G., Ci T., Yu L., Ding J. (2011). Enhancement of the fraction of the active form of an antitumor drug topotecan via an injectable hydrogel. J. Control. Release.

[86] Yang Z., Yu S., Li D., Gong Y., Zang J., Liu J., Chen X. (2018). The effect of PLGA-based hydrogel scaffold for improving the drug maximum-tolerated dose for in situ osteosarcoma treatment. Colloids Surf. B Biointerfaces.

[87] Gong C., Wang C., Wang Y., Wu Q., Zhang D., Luo F., Qian Z. (2012). Efficient inhibition of colorectal peritoneal carcinomatosis by drug loaded micelles in thermosensitive hydrogel composites. Nanoscale.

[88] Liu J., Jiang Y., Cui Y., Xu C., Ji X., Luan Y. (2014). Cytarabine-AOT catanionic vesicle-loaded biodegradable thermosensitive hydrogel as an efficient cytarabine delivery system. Int. J. Pharm..

[89] Xing Y., Chen H., Li S., Guo X. (2014). In vitro and in vivo investigation of a novel two-phase delivery system of 2-methoxyestradiol liposomes hydrogel. J. Liposome Res..

[90] Jiang L., Ding Y., Xue X., Zhou S., Li C., Zhang X., Jiang X. (2018). Entrapping multifunctional dendritic nanoparticles into a hydrogel for local therapeutic delivery and synergetic immunochemotherapy. Nano Res..

[91] Guo X., Cui F., Xing Y., Mei Q., Zhang Z. (2011). Investigation of a new injectable thermosensitive hydrogel loading solid lipid nanoparticles. Pharmazie.

[92] Ma H., He C., Cheng Y., Li D., Gong Y., Liu J., Tian H., Chen X. (2014). PLK1shRNA and doxorubicin co-loaded thermosensitive PLGA-PEG-PLGA hydrogels for osteosarcoma treatment. Biomaterials.

[93] Shen W., Chen X., Luan J., Wang D., Yu L., Ding J. (2017). Sustained Codelivery of Cisplatin and Paclitaxel via an Injectable Prodrug Hydrogel for Ovarian Cancer Treatment. ACS Appl. Mater. Interfaces.

[94] Liu Y., Xiao L., Joo K.I., Hu B., Fang J., Wang P. (2014). In situ modulation of dendritic cells by injectable thermosensitive hydrogels for cancer vaccines in mice. Biomacromolecules.

[95] Wang Y., Gong C., Yang L., Wu Q., Shi S., Shi H., Qian Z., Wei Y. (2010). 5-FU-hydrogel inhibits colorectal peritoneal carcinomatosis and tumor growth in mice. BMC Cancer.

[96] Lin X., Deng L., Xu Y., Dong A. (2012). Thermosensitive in situ hydrogel of paclitaxel conjugated poly(ε-caprolactone)-poly(ethylene glycol)-poly(ε-caprolactone). Soft Matter.

[97] Liu L., Wu Q., Ma X., Xiong D., Gong C., Qian Z., Zhao X., Wei Y. (2013). Camptothecine encapsulated composite drug delivery system for colorectal peritoneal carcinomatosis therapy: Biodegradable microsphere in thermosensitive hydrogel. Colloids Surf. B Biointerfaces.

[98] Liu M., Huang P., Wang W., Feng Z., Zhang J., Deng L., Dong A. (2019). An injectable nanocomposite hydrogel co-constructed with gold nanorods and paclitaxel-loaded nanoparticles for local chemo-photothermal synergetic cancer therapy. J. Mater. Chem. B.

[99] Peng M., Xu S., Zhang Y., Zhang L., Huang B., Fu S., Xue Z., Da Y., Dai Y., Qiao L. (2014). Thermosensitive injectable hydrogel enhances the antitumor effect of embelin in mouse hepatocellular carcinoma. J. Pharm. Sci..

[100] Wang W., Deng L., Xu S., Zhao X., Lv N., Zhang G., Gu N., Hu R., Zhang J., Liu J. (2013). A reconstituted “two into one” thermosensitive hydrogel system assembled by drug-loaded amphiphilic copolymer nanoparticles for the local delivery of paclitaxel. J. Mater. Chem. B.

[101] Huang P., Song H., Zhang Y., Liu J., Zhang J., Wang W., Li C., Kong D. (2016). Bridging the Gap between Macroscale Drug Delivery Systems and Nanomedicines: A Nanoparticle-Assembled Thermosensitive Hydrogel for Peritumoral Chemotherapy. ACS Appl. Mater. Interfaces.

[102] Huang P., Zhang Y., Wang W., Zhou J., Sun Y., Liu J., Kong D., Dong A. (2015). Co-delivery of doxorubicin and 131 I by thermosensitive micellar-hydrogel for enhanced in situ synergetic chemoradiotherapy. J. Control. Release.

[103] Zhu W., Li Y., Liu L., Chen Y., Xi F. (2012). Supramolecular hydrogels as a universal scaffold for stepwise delivering Dox and Dox/cisplatin loaded block copolymer micelles. International, J. Pharm..

[104] Ren L., He L., Sun T., Dong X., Chen Y., Huang J., Wang C. (2009). Dual-responsive supramolecular hydrogels from water-soluble PEG-grafted copolymers and cyclodextrin. Macromol. Biosci..

[105] Xu S., Wang W., Li X., Liu J., Dong A., Deng L. (2014). Sustained release of PTX-incorporated nanoparticles synergized by burst release of DOX·HCl from thermosensitive modified PEG/PCL hydrogel to improve anti-tumor efficiency. European, J. Pharm. Sci..

[106] Zhu W., Li Y., Liu L., Chen Y., Wang C., Xi F. (2010). Supramolecular hydrogels from cisplatin-loaded block copolymer nanoparticles and α-cyclodextrins with a stepwise delivery property. Biomacromolecules.

[107] Kuang H., He H., Zhang Z., Qi Y., Xie Z., Jing X., Huang Y. (2014). Injectable and biodegradable supramolecular hydrogels formed by nucleobase-terminated poly(ethylene oxide)s and α-cyclodextrin. J. Mater. Chem. B.

[108] Liu X., Li Z., Loh X.J., Chen K., Wu Y.L. (2019). Targeted and Sustained Corelease of Chemotherapeutics and Gene by Injectable Supramolecular Hydrogel for Drug-Resistant Cancer Therapy. Macromol. Rapid Commun..

[109] Shahin M., Lavasanifar A. (2010). Novel self-associating poly(ethylene oxide)-b-poly(ε-caprolactone) based drug conjugates and nano-containers for paclitaxel delivery. Int. J. Pharm..

[110] Ma G., Miao B., Song C. (2010). Thermosensitive PCL-PEG-PCL hydrogels: Synthesis, characterization, and delivery of proteins. J. Appl. Polym. Sci..

[111] Wang W., Deng L., Liu S., Li X., Zhao X., Hu R., Zhang J., Han H., Dong A. (2012). Adjustable degradation and drug release of a thermosensitive hydrogel based on a pendant cyclic ether modified poly(e-caprolactone) and poly(ethylene glycol)co-polymer. Acta. Biomater..

[112] Wang W., Song H., Zhang J., Li P., Li C., Wang C., Kong D., Zhao Q. (2015). An injectable, thermosensitive and multicompartment hydrogel for simultaneous encapsulation and independent release of a drug cocktail as an effective combination therapy platform. J. Control. Release.

[113] Yin L., Xu S., Feng Z., Deng H., Zhang J., Gao H., Deng L., Tang H., Dong A. (2017). Supramolecular hydrogel based on high-solid-content mPECT nanoparticles and cyclodextrins for local and sustained drug delivery. Biomater. Sci..

[114] Kunz-Schughart L.A., Dubrovska A., Peitzsch C., Ewe A., Aigner A., Schellenburg S., Muders M.H., Hampel S., Cirillo G., Iemma F. (2017). Nanoparticles for radiooncology: Mission, vision, challenges. Biomaterials.

[115] Peng C.L., Shih Y.H., Liang K.S., Chiang P.F., Yeh C.H., Tang I.C., Yao C.J., Lee S.Y., Luo T.Y., Shieh M.J. (2013). Development of in situ forming thermosensitive hydrogel for radiotherapy combined with chemotherapy in a mouse model of hepatocellular carcinoma. Mol. Pharm..

[116] Biolato M., Marrone G., Racco S., Di Stasi C., Miele L., Gasbarrini G., Landolfi R., Grieco A. (2010). Transarterial chemoembolization (TACE) for unresectable HCC: A new life begins?. Eur. Rev. Med. Pharmacol. Sci..

[117] Lym J.S., Nguyen Q.V., Ahn D.W., Huynh C.T., Jae H.J., Kim Y.I., Lee D.S. (2016). Sulfamethazine-based pH-sensitive hydrogels with potential application for transcatheter arterial chemoembolization therapy. Acta. Biomater..

[118] Huynh C.T., Nguyen Q.V., Lym J.S., Kim B.S., Huynh D.P., Jae H.J., Kim Y.I., Lee D.S. (2016). Intraarterial gelation of injectable cationic pH/temperature-sensitive radiopaque embolic hydrogels in a rabbit hepatic tumor model and their potential application for liver cancer treatment. RSC Adv..

[119] Gil M.S., Thambi T., Phan V.H.G., Kim S.H., Lee D.S. (2017). Injectable hydrogel-incorporated cancer cell-specific cisplatin releasing nanogels for targeted drug delivery. J. Mater. Chem. B.

[120] Varghese O.P., Liu J., Sundaram K., Hilborn J., Oommen O.P. (2016). Chondroitin sulfate derived theranostic nanoparticles for targeted drug delivery. Biomater. Sci..

[121] Andrgie A.T., Mekuria S.L., Addisu K.D., Hailemeskel B.Z., Hsu W.H., Tsai H.C., Lai J.Y. (2019). Non-Anticoagulant Heparin Prodrug Loaded Biodegradable and Injectable Thermoresponsive Hydrogels for Enhanced Anti-Metastasis Therapy. Macromol. Biosci..

[122] Phan V.H.G., Lee E., Maeng J.H., Thambi T., Kim B.S., Lee D., Lee D.S. (2016). Pancreatic cancer therapy using an injectable nanobiohybrid hydrogel. RSC Adv..

[123] Nguyen Q.V., Lym J.S., Huynh C.T., Kim B.S., Jae H.J., Kim Y.I., Lee D.S. (2016). A novel sulfamethazine-based pH-sensitive copolymer for injectable radiopaque embolic hydrogels with potential application in hepatocellular carcinoma therapy. Polym. Chem..

[124] Bobbala S., Tamboli V., McDowell A., Mitra A.K., Hook S. (2016). Novel Injectable Pentablock Copolymer Based Thermoresponsive Hydrogels for Sustained Release Vaccines. AAPS J..

[125] Kim S.H., Tan J.P.K., Fukushima K., Nederberg F., Yang Y.Y., Waymouth R.M., Hedrick J.L. (2011). Thermoresponsive nanostructured polycarbonate block copolymers as biodegradable therapeutic delivery carriers. Biomaterials.

[126] Zawaneh P.N., Singh S.P., Padera R.F., Henderson P.W., Spector J.A., Putnam D. (2010). Design of an injectable synthetic and biodegradable surgical biomaterial. Proc. Natl. Acad. Sci. USA.

[127] Lee A.L.Z., Ng V.W.L., Gao S., Hedrick J.L., Yang Y.Y. (2014). Injectable hydrogels from triblock copolymers of vitamin E-functionalized polycarbonate and poly(ethylene glycol) for subcutaneous delivery of antibodies for cancer therapy. Adv. Funct. Mater..

[128] Yang C., Lee A., Gao S., Liu S., Hedrick J.L., Yang Y.Y. (2019). Hydrogels with prolonged release of therapeutic antibody: Block junction chemistry modification of ‘ABA’ copolymers provides superior anticancer efficacy. J. Control. Release.

[129] Leprince J.G., Palin W.M., Hadis M.A., Devaux J., Leloup G. (2013). Progress in dimethacrylate-based dental composite technology and curing efficiency. Dent. Mater..

[130] Cirillo G., Spataro T., Curcio M., Spizzirri U.G., Nicoletta F.P., Picci N., Iemma F. (2015). Tunable thermo-responsive hydrogels: Synthesis, structural analysis and drug release studies. Mater. Sci. Eng. C.

[131] Pal A., Vernon B.L., Nikkhah M. (2018). Therapeutic neovascularization promoted by injectable hydrogels. Bioact. Mater..

[132] Fourniols T., Randolph L.D., Staub A., Vanvarenberg K., Leprince J.G., Préat V., Des Rieux A., Danhier F. (2015). Temozolomide-loaded photopolymerizable PEG-DMA-based hydrogel for the treatment of glioblastoma. J. Control. Release.

[133] Zhao M., Danhier F., Bastiancich C., Joudiou N., Ganipineni L.P., Tsakiris N., Gallez B., Rieux A.D., Jankovski A., Bianco J. (2018). Post-resection treatment of glioblastoma with an injectable nanomedicine-loaded photopolymerizable hydrogel induces long-term survival. Int. J. Pharm..

[134] Zhang H., Zhu X., Ji Y., Jiao X., Chen Q., Hou L., Zhang Z. (2015). Near-infrared-triggered in situ hybrid hydrogel system for synergistic cancer therapy. J. Mater. Chem. B.

[135] Wu H., Song L., Chen L., Huang Y., Wu Y., Zang F., An Y., Lyu H., Ma M., Chen J. (2017). Injectable thermosensitive magnetic nanoemulsion hydrogel for multimodal-imaging-guided accurate thermoablative cancer therapy. Nanoscale.

[136] Khang M.K., Zhou J., Huang Y., Hakamivala A., Tang L. (2018). Preparation of a novel injectable in situ-gelling nanoparticle with applications in controlled protein release and cancer cell entrapment. RSC Adv..

[137] Ishii S., Kaneko J., Nagasaki Y. (2016). Development of a long-acting, protein-loaded, redox-active, injectable gel formed by a polyion complex for local protein therapeutics. Biomaterials.

[138] Xu X., Huang Z., Zhang X., He S., Sun X., Shen Y., Yan M., Zhao C. (2017). Injectable, NIR/pH-Responsive Nanocomposite Hydrogel as Long-Acting Implant for Chemophotothermal Synergistic Cancer Therapy. ACS Appl. Mater. Interfaces.

[139] Wu Y., Wang H., Gao F., Xu Z., Dai F., Liu W. (2018). An Injectable Supramolecular Polymer Nanocomposite Hydrogel for Prevention of Breast Cancer Recurrence with Theranostic and Mammoplastic Functions. Adv. Funct. Mater..

[140] Song Z., Han Z., Lv S., Chen C., Chen L., Yin L., Cheng J. (2017). Synthetic polypeptides: From polymer design to supramolecular assembly and biomedical application. Chem. Soc. Rev..

[141] Shen Y., Fu X., Fu W., Li Z. (2015). Biodegradable stimuli-responsive polypeptide materials prepared by ring opening polymerization. Chem. Soc. Rev..

[142] Deming T.J. (2016). Synthesis of Side-Chain Modified Polypeptides. Chem. Rev..

[143] Maude S., Ingham E., Aggeli A. (2013). Biomimetic self-assembling peptides as scaffolds for soft tissue engineering. Nanomedicine.

[144] Szkolar L., Guilbaud J.B., Miller A.F., Gough J.E., Saiani A. (2014). Enzymatically triggered peptide hydrogels for 3D cell encapsulation and culture. J. Pept. Sci..

[145] Rodriguez A.L., Wang T.Y., Bruggeman K.F., Li R., Williams R.J., Parish C.L., Nisbet D.R. (2016). Tailoring minimalist self-assembling peptides for localized viral vector gene delivery. Nano Res..

[146] Abbas M., Xing R., Zhang N., Zou Q., Yan X. (2018). Antitumor Photodynamic Therapy Based on Dipeptide Fibrous Hydrogels with Incorporation of Photosensitive Drugs. ACS Biomater. Sci. Eng..

[147] Xing R., Li S., Zhang N., Shen G., Möhwald H., Yan X. (2017). Self-Assembled Injectable Peptide Hydrogels Capable of Triggering Antitumor Immune Response. Biomacromolecules.

[148] Weiden J., Voerman D., Dölen Y., Das R.K., Van Duffelen A., Hammink R., Eggermont L.J., Rowan A.E., Tel J., Figdor C.G. (2018). Injectable biomimetic hydrogels as tools for efficient T Cell expansion and delivery. Front. Immunol..

[149] Yamada Y., Chowdhury A., Schneider J.P., Stetler-Stevenson W.G. (2018). Macromolecule-Network Electrostatics Controlling Delivery of the Biotherapeutic Cell Modulator TIMP-2. Biomacromolecules.

[150] Qi Y., Min H., Mujeeb A., Zhang Y., Han X., Zhao X., Anderson G.J., Zhao Y., Nie G. (2018). Injectable Hexapeptide Hydrogel for Localized Chemotherapy Prevents Breast Cancer Recurrence. ACS Appl. Mater. Interfaces.

[151] Mei L., Xu K., Zhai Z., He S., Zhu T., Zhong W. (2019). Doxorubicin-reinforced supramolecular hydrogels of RGD-derived peptide conjugates for pH-responsive drug delivery. Org. Biomol. Chem..

[152] Leach D.G., Dharmaraj N., Piotrowski S.L., Lopez-Silva T.L., Lei Y.L., Sikora A.G., Young S., Hartgerink J.D. (2018). STINGel: Controlled release of a cyclic dinucleotide for enhanced cancer immunotherapy. Biomaterials.

[153] Jin H., Wan C., Zou Z., Zhao G., Zhang L., Geng Y., Chen T., Huang A., Jiang F., Feng J.P. (2018). Tumor Ablation and Therapeutic Immunity Induction by an Injectable Peptide Hydrogel. ACS Nano.

[154] Hu C., Liu X., Ran W., Meng J., Zhai Y., Zhang P., Yin Q., Yu H., Zhang Z., Li Y. (2017). Regulating cancer associated fibroblasts with losartan-loaded injectable peptide hydrogel to potentiate chemotherapy in inhibiting growth and lung metastasis of triple negative breast cancer. Biomaterials.

[155] Li L., Gu J., Zhang J., Xie Z., Lu Y., Shen L., Dong Q., Wang Y. (2015). Injectable and biodegradable pH-responsive hydrogels for localized and sustained treatment of human fibrosarcoma. ACS Appl. Mater. Interfaces.

[156] Yu S., Zhang D., He C., Sun W., Cao R., Cui S., Deng M., Gu Z., Chen X. (2017). Injectable Thermosensitive Polypeptide-Based CDDP-Complexed Hydrogel for Improving Localized Antitumor Efficacy. Biomacromolecules.

[157] Song H., Huang P., Niu J., Shi G., Zhang C., Kong D., Wang W. (2018). Injectable polypeptide hydrogel for dual-delivery of antigen and TLR3 agonist to modulate dendritic cells in vivo and enhance potent cytotoxic T-lymphocyte response against melanoma. Biomaterials.

[158] Wei L., Chen J., Zhao S., Ding J., Chen X. (2017). Thermo-sensitive polypeptide hydrogel for locally sequential delivery of two-pronged antitumor drugs. Acta. Biomater..

[159] Cheng Y., He C., Ding J., Xiao C., Zhuang X., Chen X. (2013). Thermosensitive hydrogels based on polypeptides for localized and sustained delivery of anticancer drugs. Biomaterials.

[160] Wu C., Li R., Yin Y., Wang J., Zhang L., Zhong W. (2017). Redox-responsive supramolecular hydrogel based on 10-hydroxy camptothecin-peptide covalent conjugates with high loading capacity for drug delivery. Mater. Sci. Eng. C.

[161] Wang H., Lv L., Xu G., Yang C., Sun J., Yang Z. (2012). Molecular hydrogelators consist of Taxol and short peptides/amino acids. J. Mater. Chem..

[162] Singh M., Kundu S., Reddy M.A., Sreekanth V., Motiani R.K., Sengupta S., Srivastava A., Bajaj A. (2014). Injectable small molecule hydrogel as a potential nanocarrier for localized and sustained in vivo delivery of doxorubicin. Nanoscale.

[163] Lin Q., Yang Y., Hu Q., Guo Z., Liu T., Xu J., Wu J., Kirk T.B., Ma D., Xue W. (2017). Injectable supramolecular hydrogel formed from α-cyclodextrin and PEGylated arginine-functionalized poly(L-lysine) dendron for sustained MMP-9 shRNA plasmid delivery. Acta. Biomater..

[164] Ma Y., Fu X., Shen Y., Fu W., Li Z. (2014). Irreversible low critical solution temperature behaviors of thermal-responsive OEGylated poly(L-cysteine) containing disulfide bonds. Macromolecules.

[165] Zhang S., Fu W., Li Z. (2014). Supramolecular hydrogels assembled from nonionic poly(ethylene glycol)-b-polypeptide diblocks containing OEGylated poly-l-glutamate. Polym. Chem..

[166] Singh S.K., Singh S., Wlillard J., Singh R. (2017). Drug delivery approaches for breast cancer. Int. J. Nanomed..

[167] Kesharwani P., Gothwal A., Iyer A.K., Jain K., Chourasia M.K., Gupta U. (2018). Dendrimer nanohybrid carrier systems: An expanding horizon for targeted drug and gene delivery. Drug Discov. Today.

[168] Kitchens K.M., El-Sayed M.E.H., Ghandehari H. (2005). Transepithelial and endothelial transport of poly (amidoamine) dendrimers. Adv. Drug Deliv. Rev..

[169] Northfelt D.W., Dezube B.J., Thommes J.A., Miller B.J., Fischl M.A., Friedman-Kien A., Kaplan L.D., Du Mond C., Mamelok R.D., Henry D.H. (1998). Pegylated-liposomal doxorubicin versus doxorubicin, bleomycin, and vincristine in the treatment of AIDS-related Kaposi’s sarcoma: Results of a randomized phase III clinical trial. J. Clin. Oncol..

[170] Lo Y.W., Sheu M.T., Chiang W.H., Chiu Y.L., Tu C.M., Wang W.Y., Wu M.H., Wang Y.C., Lu M., Ho H.O. (2019). In situ chemically crosslinked injectable hydrogels for the subcutaneous delivery of trastuzumab to treat breast cancer. Acta. Biomater..

[171] Zhang H., Zhao C., Cao H., Wang G., Song L., Niu G., Yang H., Ma J., Zhu S. (2010). Hyperbranched poly(amine-ester) based hydrogels for controlled multi-drug release in combination chemotherapy. Biomaterials.

[172] Xu L., Cooper R.C., Wang J., Yeudall W.A., Yang H. (2017). Synthesis and Application of Injectable Bioorthogonal Dendrimer Hydrogels for Local Drug Delivery. ACS Biomater. Sci. Eng..

[173] Patil S.S., Shinde V.S., Misra R.D.K. (2018). pH and reduction dual-stimuli-responsive PEGDA/PAMAM injectable network hydrogels via aza-michael addition for anticancer drug delivery. J. Polym. Sci. A Polym. Chem..

[174] Yang W.J., Zhou P., Liang L., Cao Y., Qiao J., Li X., Teng Z., Wang L. (2018). Nanogel-Incorporated Injectable Hydrogel for Synergistic Therapy Based on Sequential Local Delivery of Combretastatin-A4 Phosphate (CA4P) and Doxorubicin (DOX). ACS Appl. Mater. Interfaces.

[175] Kharkar P.M., Kloxin A.M., Kiick K.L. (2014). Dually degradable click hydrogels for controlled degradation and protein release. J. Mater. Chem. B.

[176] Huang Z., Delparastan P., Burch P., Cheng J., Cao Y., Messersmith P.B. (2018). Injectable dynamic covalent hydrogels of boronic acid polymers cross-linked by bioactive plant-derived polyphenols. Biomater. Sci..

[177] Gao W., Liang Y., Peng X., Hu Y., Zhang L., Wu H., He B. (2016). In situ injection of phenylboronic acid based low molecular weight gels for efficient chemotherapy. Biomaterials.

[178] Seib F.P., Tsurkan M., Freudenberg U., Kaplan D.L., Werner C. (2016). Heparin-Modified Polyethylene Glycol Microparticle Aggregates for Focal Cancer Chemotherapy. ACS Biomater. Sci. Eng..

[179] Fang Y., Xue J., Gao S., Lu A., Yang D., Jiang H., He Y., Shi K. (2017). Cleavable PEGylation: A strategy for overcoming the “PEG dilemma” in efficient drug delivery. Drug Deliv..

[180] Bastiancich C., Bianco J., Vanvarenberg K., Ucakar B., Joudiou N., Gallez B., Bastiat G., Lagarce F., Préat V., Danhier F. (2017). Injectable nanomedicine hydrogel for local chemotherapy of glioblastoma after surgical resection. J. Control. Release.

[181] Ye Y., Hu X. (2016). A pH-sensitive injectable nanoparticle composite hydrogel for anticancer drug delivery. J. Nanomater..

[182] Makharza S., Cirillo G., Bachmatiuk A., Ibrahim I., Ioannides N., Trzebicka B., Hampel S., Ruemmeli M.H. (2013). Graphene oxide-based drug delivery vehicles: Functionalization, characterization, and cytotoxicity evaluation. J. Nanoparticle Res..

[183] Vittorio O., Le Grand M., Makharza S.A., Curcio M., Tucci P., Iemma F., Nicoletta F.P., Hampel S., Cirillo G. (2018). Doxorubicin synergism and resistance reversal in human neuroblastoma BE(2)C cell lines: An in vitro study with dextran-catechin nanohybrids. Eur. J. Pharm. Biopharm..

[184] Lerra L., Farfalla A., Sanz B., Cirillo G., Vittorio O., Voli F., Grand M.L., Curcio M., Nicoletta F.P., Dubrovska A. (2019). Graphene oxide functional nanohybrids with magnetic nanoparticles for improved vectorization of doxorubicin to neuroblastoma cells. Pharmaceutics.

[185] Thambi T., Phan V.H.G., Lee D.S. (2016). Stimuli-Sensitive Injectable Hydrogels Based on Polysaccharides and Their Biomedical Applications. Macromol. Rapid Commun..

[186] Spizzirri U.G., Altimari I., Puoci F., Parisi O.I., Iemma F., Picci N. (2011). Innovative antioxidant thermo-responsive hydrogels by radical grafting of catechin on inulin chain. Carbohydr. Polym..

[187] Ahsan S.M., Thomas M., Reddy K.K., Sooraparaju S.G., Asthana A., Bhatnagar I. (2018). Chitosan as biomaterial in drug delivery and tissue engineering. Int. J. Biol. Macromol..

[188] Kozen B.G., Kircher S.J., Henao J., Godinez F.S., Johnson A.S. (2008). An alternative hemostatic dressing: Comparison of CELOX, HemCon, and QuikClot. Acad. Emerg. Med..

[189] Ueno H., Mori T., Fujinaga T. (2001). Topical formulations and wound healing applications of chitosan. Adv. Drug Deliv. Rev..

[190] Karimi A.R., Khodadadi A., Hadizadeh M. (2016). A nanoporous photosensitizing hydrogel based on chitosan cross-linked by zinc phthalocyanine: An injectable and pH-stimuli responsive system for effective cancer therapy. RSC Adv..

[191] Abdel-Bar H.M., Abdel-Reheem A.Y., Osman R., Awad G.A.S., Mortada N. (2014). Defining cisplatin incorporation properties in thermosensitive injectable biodegradable hydrogel for sustained delivery and enhanced cytotoxicity. Int. J. Pharm..

[192] Fathi M., Alami-Milani M., Geranmayeh M.H., Barar J., Erfan-Niya H., Omidi Y. (2019). Dual thermo-and pH-sensitive injectable hydrogels of chitosan/(poly(N-isopropylacrylamide-co-itaconic acid)) for doxorubicin delivery in breast cancer. Int. J. Biol. Macromol..

[193] Zhang W., Jin X., Li H., Zhang R.R., Wu C.W. (2018). Injectable and body temperature sensitive hydrogels based on chitosan and hyaluronic acid for pH sensitive drug release. Carbohydr. Polym..

[194] Saeednia L., Yao L., Cluff K., Asmatulu R. (2019). Sustained Releasing of Methotrexate from Injectable and Thermosensitive Chitosan-Carbon Nanotube Hybrid Hydrogels Effectively Controls Tumor Cell Growth. ACS Omega.

[195] Huang F.Y.J., Hung C.C., Chang C.W., Chao J.H., Hsieh B.T. (2018). Evaluation of injectable chitosan-based co-crosslinking hydrogel for local delivery of 188Re-LIPO-DOX to breast-tumor-bearing mouse model. Anticancer. Res..

[196] Alexander A., Ajazuddin A., Khan J., Saraf S. (2014). Formulation and evaluation of chitosan-based long-acting injectable hydrogel for PEGylated melphalan conjugate. J. Pharm. Pharmacol..

[197] López-Noriega A., Hastings C.L., Ozbakir B., O’Donnell K.E., O’Brien F.J., Storm G., Hennink W.E., Duffy G.P., Ruiz-Hernández E. (2014). Hyperthermia-Induced Drug Delivery from Thermosensitive Liposomes Encapsulated in an Injectable Hydrogel for Local Chemotherapy. Adv. Healthc. Mater..

[198] Xing J., Qi X., Jiang Y., Zhu X., Zhang Z., Qin X., Wu Z. (2015). Topotecan hydrochloride liposomes incorporated into thermosensitive hydrogel for sustained and efficient in situ therapy of H22 tumor in Kunming mice. Pharm. Dev. Technol..

[199] Huang F.Y.J., Gan G.Y., Lin W.Y., Huang L.K., Luo T.Y., Hong J.J., Hsieh B.T. (2014). Investigation of the local delivery of an intelligent chitosan-based 188Re thermosensitive in situ-forming hydrogel in an orthotopic hepatoma-bearing rat model. J. Radioanal. Nucl. Chem..

[200] Zhang D., Sun P., Li P., Xue A., Zhang X., Zhang H., Jin X. (2013). A magnetic chitosan hydrogel for sustained and prolonged delivery of Bacillus Calmette-Guérin in the treatment of bladder cancer. Biomaterials.

[201] Le Renard P.E., Jordan O., Faes A., Petri-Fink A., Hofmann H., Rüfenacht D., Bosman F., Buchegger F., Doelker E. (2010). The in vivo performance of magnetic particle-loaded injectable, in situ gelling, carriers for the delivery of local hyperthermia. Biomaterials.

[202] Zhu X., Zhang H., Huang H., Zhang Y., Hou L., Zhang Z. (2015). Functionalized graphene oxide-based thermosensitive hydrogel for magnetic hyperthermia therapy on tumors. Nanotechnology.

[203] Saeednia L., Yao L., Berndt M., Cluff K., Asmatulu R. (2017). Structural and biological properties of thermosensitive chitosan–graphene hybrid hydrogels for sustained drug delivery applications. J. Biomed. Mater. Res. A.

[204] Fletcher N.A., Krebs M.D. (2018). Sustained delivery of anti-VEGF from injectable hydrogel systems provides a prolonged decrease of endothelial cell proliferation and angiogenesis: In vitro. RSC Adv..

[205] Chen C.H., Kuo C.Y., Chen S.H., Mao S.H., Chang C.Y., Shalumon K.T., Chen J.P. (2018). Thermosensitive injectable hydrogel for simultaneous intraperitoneal delivery of doxorubicin and prevention of peritoneal adhesion. Int. J. Mol. Sci..

[206] Fong Y.T., Chen C.H., Chen J.P. (2017). Intratumoral delivery of doxorubicin on folate-conjugated graphene oxide by in-situ forming thermo-sensitive hydrogel for breast cancer therapy. Nanomaterials.

[207] Hyun H., Park M.H., Lim W., Kim S.Y., Jo D., Jung J.S., Jo G., Um S., Lee D.W., Yang D.H. (2018). Injectable visible light-cured glycol chitosan hydrogels with controlled release of anticancer drugs for local cancer therapy in vivo: A feasible study. Artif. Cells Nanomed. Biotechnol..

[208] Zhou X., Li Y., Chen S., Fu Y.N., Wang S., Li G., Tao L., Wei Y., Wang X., Liang J.F. (2018). Dynamic agent of an injectable and self-healing drug-loaded hydrogel for embolization therapy. Colloids Surf. B Biointerfaces.

[209] Xia L.Y., Zhang X., Cao M., Chen Z., Wu F.G. (2017). Enhanced Fluorescence Emission and Singlet Oxygen Generation of Photosensitizers Embedded in Injectable Hydrogels for Imaging-Guided Photodynamic Cancer Therapy. Biomacromolecules.

[210] Xie W., Gao Q., Guo Z., Wang D., Gao F., Wang X., Wei Y., Zhao L. (2017). Injectable and self-healing thermosensitive magnetic hydrogel for asynchronous control release of doxorubicin and docetaxel to treat triple-negative breast cancer. ACS Appl. Mater. Interfaces.

[211] Liu Z., Xu G., Wang C., Li C., Yao P. (2017). Shear-responsive injectable supramolecular hydrogel releasing doxorubicin loaded micelles with pH-sensitivity for local tumor chemotherapy. Int. J. Pharm..

[212] Wang Q.Q., Kong M., An Y., Liu Y., Li J.J., Zhou X., Feng C., Li J., Jiang S.Y., Cheng X.J. (2013). Hydroxybutyl chitosan thermo-sensitive hydrogel: A potential drug delivery system. J. Mater. Sci..

[213] Khan S., Akhtar N., Minhas M.U., Badshah S.F. (2019). pH/Thermo-Dual Responsive Tunable In Situ Cross-Linkable Depot Injectable Hydrogels Based on Poly(N-Isopropylacrylamide)/Carboxymethyl Chitosan with Potential of Controlled Localized and Systemic Drug Delivery. AAPS Pharm. Sci. Tech..

[214] Wang H., Song F., Chen Q., Hu R., Jiang Z., Yang Y., Han B. (2015). Antitumor and antimetastasis effects of macerating solutions from an injectable chitosan-based hydrogel on hepatocarcinoma. J. Biomed. Mater. Res.-A.

[215] Qu J., Zhao X., Ma P.X., Guo B. (2017). pH-responsive self-healing injectable hydrogel based on N-carboxyethyl chitosan for hepatocellular carcinoma therapy. Acta. Biomater..

[216] Chen X., Fan M., Tan H., Ren B., Yuan G., Jia Y., Li J., Xiong D., Xing X., Niu X. (2019). Magnetic and self-healing chitosan-alginate hydrogel encapsulated gelatin microspheres via covalent cross-linking for drug delivery. Mater. Sci. Eng. C.

[217] Qian C., Zhang T., Gravesande J., Baysah C., Song X., Xing J. (2019). Injectable and self-healing polysaccharide-based hydrogel for pH-responsive drug release. Int. J. Biol. Macromol..

[218] Gao N., Lü S., Gao C., Wang X., Xu X., Bai X., Feng C., Liu M. (2016). Injectable shell-crosslinked F127 micelle/hydrogel composites with pH and redox sensitivity for combined release of anticancer drugs. Chem. Eng. J..

[219] Li J., Hu W., Zhang Y., Tan H., Yan X., Zhao L., Liang H. (2015). PH and glucose dually responsive injectable hydrogel prepared by in situ crosslinking of phenylboronic modified chitosan and oxidized dextran. J. Polym. Sci. A Polym. Chem..

[220] Liang Y., Zhao X., Ma P.X., Guo B., Du Y., Han X. (2019). pH-responsive injectable hydrogels with mucosal adhesiveness based on chitosan-grafted-dihydrocaffeic acid and oxidized pullulan for localized drug delivery. J. Colloid Interface Sci..

[221] Jalalvandi E., Shavandi A. (2018). In situ-forming and pH-responsive hydrogel based on chitosan for vaginal delivery of therapeutic agents. J. Mater. Sci. Mater. Med..

[222] Shi J., Guobao W., Chen H., Zhong W., Qiu X., Xing M.M.Q. (2014). Schiff based injectable hydrogel for in situ pH-triggered delivery of doxorubicin for breast tumor treatment. Polym. Chem..

[223] Zahedi P., De Souza R., Piquette-Miller M., Allen C. (2011). Docetaxel distribution following intraperitoneal administration in mice. Journal of pharmacy & pharmaceutical sciences: A publication of the Canadian Society for Pharmaceutical Sciences. J. Pharm. Pharm. Sci..

[224] Yavvari P.S., Pal S., Kumar S., Kar A., Awasthi A.K., Naaz A., Srivastava A., Bajaj A. (2017). Injectable, Self-Healing Chimeric Catechol-Fe(III) Hydrogel for Localized Combination Cancer Therapy. ACS Biomater. Sci. Eng..

[225] Belali S., Karimi A.R., Hadizadeh M. (2018). Cell-specific and pH-sensitive nanostructure hydrogel based on chitosan as a photosensitizer carrier for selective photodynamic therapy. Int. J. Biol. Macromol..

[226] Ning P., Lü S., Bai X., Wu X., Gao C., Wen N., Liu M. (2018). High encapsulation and localized delivery of curcumin from an injectable hydrogel. Mater. Sci. Eng. C.

[227] Burdick J.A. (2012). Injectable gels for tissue/organ repair. Biomed. Mater..

[228] Seliktar D. (2012). Designing cell-compatible hydrogels for biomedical applications. Science.

[229] Mitragotri S., Burke P.A., Langer R. (2014). Overcoming the challenges in administering biopharmaceuticals: Formulation and delivery strategies. Nat. Rev. Drug Discov..

[230] Burdick J.A., Prestwich G.D. (2011). Hyaluronic acid hydrogels for biomedical applications. Adv. Mater..

[231] Chen Y.Y., Wu H.C., Sun J.S., Dong G.C., Wang T.W. (2013). Injectable and thermoresponsive self-assembled nanocomposite hydrogel for long-term anticancer drug delivery. Langmuir.

[232] Zhao Y., Yan H., Qiao S., Zhang L., Wang T., Meng Q., Chen X., Lin F.H., Guo K., Li C. (2016). Hydrogels bearing bioengineered mimetic embryonic microenvironments for tumor reversion. J. Mater. Chem. B.

[233] Ohta S., Hiramoto S., Amano Y., Emoto S., Yamaguchi H., Ishigami H., Kitayama J., Ito T. (2017). Intraperitoneal Delivery of Cisplatin via a Hyaluronan-Based Nanogel/in Situ Cross-Linkable Hydrogel Hybrid System for Peritoneal Dissemination of Gastric Cancer. Mol. Pharm..

[234] Xu K., Lee F., Gao S.J., Chung J.E., Yano H., Kurisawa M. (2013). Injectable hyaluronic acid-tyramine hydrogels incorporating interferon-α2a for liver cancer therapy. J. Control. Release.

[235] Xu K., Lee F., Gao S., Tan M.H., Kurisawa M. (2015). Hyaluronidase-incorporated hyaluronic acid-tyramine hydrogels for the sustained release of trastuzumab. J. Control. Release.

[236] Ueda K., Akiba J., Ogasawara S., Todoroki K., Nakayama M., Sumi A., Kusano H., Sanada S., Suekane S., Xu K. (2016). Growth inhibitory effect of an injectable hyaluronic acid-tyramine hydrogels incorporating human natural interferon-α and sorafenib on renal cell carcinoma cells. Acta. Biomater..

[237] He M., Sui J., Chen Y., Bian S., Cui Y., Zhou C., Sun Y., Liang J., Fan Y., Zhang X. (2017). Localized multidrug co-delivery by injectable self-crosslinking hydrogel for synergistic combinational chemotherapy. J. Mater. Chem. B.

[238] Chen X., Liu Z. (2016). A pH-Responsive Hydrogel Based on a Tumor-Targeting Mesoporous Silica Nanocomposite for Sustained Cancer Labeling and Therapy. Macromol. Rapid Commun..

[239] Chen X., Liu Z., Parker S.G., Zhang X., Gooding J.J., Ru Y., Liu Y., Zhou Y. (2016). Light-Induced Hydrogel Based on Tumor-Targeting Mesoporous Silica Nanoparticles as a Theranostic Platform for Sustained Cancer Treatment. ACS Appl. Mater. Interfaces.

[240] Ranga A., Lutolf M.P., Hilborn J., Ossipov D.A. (2016). Hyaluronic Acid Hydrogels Formed in Situ by Transglutaminase-Catalyzed Reaction. Biomacromolecules.

[241] Moon R.J., Martini A., Nairn J., Simonsen J., Youngblood J. (2011). Cellulose nanomaterials review: Structure, properties and nanocomposites. Chem. Soc. Rev..

[242] Ngwabebhoh F.A., Yildiz U. (2019). Nature-derived fibrous nanomaterial toward biomedicine and environmental remediation: Today’s state and future prospects. J. Appl. Polym. Sci..

[243] You J., Cao J., Zhao Y., Zhang L., Zhou J., Chen Y. (2016). Improved Mechanical Properties and Sustained Release Behavior of Cationic Cellulose Nanocrystals Reinforeced Cationic Cellulose Injectable Hydrogels. Biomacromolecules.

[244] Ding L., Wang Q., Shen M., Sun Y., Zhang X., Huang C., Chen J., Li R., Duan Y. (2017). Thermoresponsive nanocomposite gel for local drug delivery to suppress the growth of glioma by inducing autophagy. Autophagy.

[245] Xing C., Chen S., Qiu M., Liang X., Liu Q., Zou Q., Li Z., Xie Z., Wang D., Dong B. (2018). Conceptually Novel Black Phosphorus/Cellulose Hydrogels as Promising Photothermal Agents for Effective Cancer Therapy. Adv. Healthc. Mater..

[246] Yang J.S., Xie Y.J., He W. (2011). Research progress on chemical modification of alginate: A review. Carbohydr. Polym..

[247] Wróblewska-Krepsztul J., Rydzkowski T., Michalska-Pożoga I., Thakur V.K. (2019). Biopolymers for biomedical and pharmaceutical applications: Recent advances and overview of alginate electrospinning. Nanomaterials.

[248] Wang C., Wang X., Dong K., Luo J., Zhang Q., Cheng Y. (2016). Injectable and responsively degradable hydrogel for personalized photothermal therapy. Biomaterials.

[249] Chalanqui M.J., Pentlavalli S., McCrudden C., Chambers P., Ziminska M., Dunne N., McCarthy H.O. (2019). Influence of alginate backbone on efficacy of thermo-responsive alginate-g-P(NIPAAm) hydrogel as a vehicle for sustained and controlled gene delivery. Mater. Sci. Eng. C.

[250] Liu M., Song X., Wen Y., Zhu J.L., Li J. (2017). Injectable Thermoresponsive Hydrogel Formed by Alginate-g-Poly(N-isopropylacrylamide) That Releases Doxorubicin-Encapsulated Micelles as a Smart Drug Delivery System. ACS Appl. Mater. Interfaces.

[251] Davoodi P., Ng W.C., Srinivasan M.P., Wang C.H. (2017). Codelivery of anti-cancer agents via double-walled polymeric microparticles/injectable hydrogel: A promising approach for treatment of triple negative breast cancer. Biotechnol. Bioeng..

[252] Davoodi P., Ng W.C., Yan W.C., Srinivasan M.P., Wang C.H. (2016). Double-walled microparticles-embedded self-cross-linked, injectable, and antibacterial hydrogel for controlled and sustained release of chemotherapeutic agents. ACS Appl. Mater. Interfaces.

[253] Liu J., Qi C., Tao K., Zhang J., Xu L., Jiang X., Zhang Y., Huang L., Li Q., Xie H. (2016). Sericin/Dextran Injectable Hydrogel as an Optically Trackable Drug Delivery System for Malignant Melanoma Treatment. ACS Appl. Mater. Interfaces.

[254] Li L., Wang C., Huang Q., Xiao J., Zhang Q., Cheng Y. (2018). A degradable hydrogel formed by dendrimer-encapsulated platinum nanoparticles and oxidized dextran for repeated photothermal cancer therapy. J. Mater. Chem. B.

[255] Deng J., Xun X., Zheng W., Su Y., Zheng L., Wang C., Su M. (2018). Sequential delivery of bismuth nanoparticles and doxorubicin by injectable macroporous hydrogels for combined anticancer kilovoltage X-ray radio- and chemo-therapy. J. Mater. Chem. B.

[256] GuhaSarkar S., More P., Banerjee R. (2017). Urothelium-adherent, ion-triggered liposome-in-gel system as a platform for intravesical drug delivery. J. Control. Release.

[257] Zheng Y., Liang Y., Zhang D., Zhou Z., Li J., Sun X., Liu Y.N. (2018). Fabrication of injectable CuS nanocomposite hydrogels based on UCST-type polysaccharides for NIR-triggered chemo-photothermal therapy. Chem. Commun..

[258] Hou M., Yang R., Zhang L., Liu G., Xu Z., Kang Y., Xue P. (2018). Injectable and Natural Humic Acid/Agarose Hybrid Hydrogel for Localized Light-Driven Photothermal Ablation and Chemotherapy of Cancer. ACS Biomater. Sci. Eng..

[259] Niu X., Zhang Z., Zhong Y. (2017). Hydrogel loaded with self-assembled dextran sulfate-doxorubicin complexes as a delivery system for chemotherapy. Mater. Sci. Eng. C.

[260] Nguyen K., Dang P.N., Alsberg E. (2013). Functionalized, biodegradable hydrogels for control over sustained and localized siRNA delivery to incorporated and surrounding cells. Acta. Biomater..

[261] Vittorio O., Cirillo G., Iemma F., Di Turi G., Jacchetti E., Curcio M., Barbuti S., Funel N., Parisi O.I., Puoci F. (2012). Dextran-catechin conjugate: A potential treatment against the pancreatic ductal adenocarcinoma. Pharm. Res..

[262] Vittorio O., Brandl M., Cirillo G., Kimpton K., Hinde E., Gaus K., Yee E., Kumar N., Duong H., Fleming C. (2016). Dextran-Catechin: An anticancer chemically-modified natural compound targeting copper that attenuates neuroblastoma growth. Oncotarget.

[263] Agarwal A., Gupta U., Asthana A., Jain N.K. (2009). Dextran conjugated dendritic nanoconstructs as potential vectors for anti-cancer agent. Biomaterials.

[264] Abdo Qasem A.A., Alamri M.S., Mohamed A.A., Hussain S., Mahmood K., Ibraheem M.A. (2017). High Soluble-Fiber Pudding: Formulation, Processing, Texture and Sensory Properties. J. Food Process. Preserv..

[265] Carlini A.S., Gaetani R., Braden R.L., Luo C., Christman K.L., Gianneschi N.C. (2019). Enzyme-responsive progelator cyclic peptides for minimally invasive delivery to the heart post-myocardial infarction. Nat. Commun..

[266] Haines-Butterick L., Rajagopal K., Branco M., Salick D., Rughani R., Pilarz M., Lamm M.S., Pochan D.J., Schneider J.P. (2007). Controlling hydrogelation kinetics by peptide design for three-dimensional encapsulation and injectable delivery of cells. Proc. Natl. Acad. Sci. USA.

[267] Mano J.F. (2008). Stimuli-responsive polymeric systems for biomedical applications. Adv. Eng. Mater..

[268] Xing R., Liu K., Jiao T., Zhang N., Ma K., Zhang R., Zou Q., Ma G., Yan X. (2016). An Injectable Self-Assembling Collagen-Gold Hybrid Hydrogel for Combinatorial Antitumor Photothermal/Photodynamic Therapy. Adv. Mater..

[269] Upadhyay A., Kandi R., Rao C.P. (2018). Injectable, Self-Healing, and Stress Sustainable Hydrogel of BSA as a Functional Biocompatible Material for Controlled Drug Delivery in Cancer Cells. ACS Sustain. Chem. Eng..

[270] Kim I., Choi J.S., Lee S., Byeon H.J., Lee E.S., Shin B.S., Choi H.G., Lee K.C., Youn Y.S. (2015). In situ facile-forming PEG cross-linked albumin hydrogels loaded with an apoptotic TRAIL protein. J. Control. Release.

[271] Qian H.Q., Qian K.Y., Cai J., Yang Y., Zhu L.J., Liu B.R. (2019). Therapy for Gastric Cancer with Peritoneal Metastasis Using Injectable Albumin Hydrogel Hybridized with Paclitaxel-Loaded Red Blood Cell Membrane Nanoparticles. ACS Biomater. Sci. Eng..

[272] Curcio M., Altimari I., Spizzirri U.G., Cirillo G., Vittorio O., Puoci F., Picci N., Iemma F. (2013). Biodegradable gelatin-based nanospheres as pH-responsive drug delivery systems. J. Nanoparticle Res..

[273] Curcio M., Spizzirri U.G., Iemma F., Puoci F., Cirillo G., Parisi O.I., Picci N. (2010). Grafted thermo-responsive gelatin microspheres as delivery systems in triggered drug release. Eur. J. Pharm. Biopharm..

[274] Oh E., Oh J.E., Hong J., Chung Y., Lee Y., Park K.D., Kim S., Yun C.O. (2017). Optimized biodegradable polymeric reservoir-mediated local and sustained co-delivery of dendritic cells and oncolytic adenovirus co-expressing IL-12 and GM-CSF for cancer immunotherapy. J. Control. Release.

[275] Takei T., Sugihara K., Yoshida M., Kawakami K. (2013). Injectable and biodegradable sugar beet pectin/gelatin hydrogels for biomedical applications. J. Biomater. Sci. Polym. Ed..

[276] Ciobanu B.C., Cadinoiu A.N., Popa M., Desbrières J., Peptu C.A. (2014). Modulated release from liposomes entrapped in chitosan/gelatin hydrogels. Mater. Sci. Eng. C.

[277] Franke K., Baur M., Daum L., Vaegler M., Sievert K.D., Schlosshauer B. (2013). Prostate carcinoma cell growth-inhibiting hydrogel supports axonal regeneration in vitro. Neurosci. Lett..

[278] Cirillo G., Vittorio O., Hampel S., Spizzirri U.G., Picci N., Iemma F. (2013). Incorporation of carbon nanotubes into a gelatin-catechin conjugate: Innovative approach for the preparation of anticancer materials. Int. J. Pharm..

[279] Zhou M., Liu S., Jiang Y., Ma H., Shi M., Wang Q., Zhong W., Liao W., Xing M.M.Q. (2015). Doxorubicin-Loaded Single Wall Nanotube Thermo-Sensitive Hydrogel for Gastric Cancer Chemo-Photothermal Therapy. Adv. Funct. Mater..

[280] Cirillo G., Hampel S., Spizzirri U.G., Parisi O.I., Picci N., Iemma F. (2014). Carbon Nanotubes Hybrid Hydrogels in Drug Delivery: A Perspective Review. Biomed. Res. Int..

[281] Cirillo G., Caruso T., Hampel S., Haase D., Puoci F., Ritschel M., Leonhardt A., Curcio M., Iemma F., Khavrus V. (2013). Novel carbon nanotube composites by grafting reaction with water-compatible redox initiator system. Colloid Polym. Sci..

[282] He G., Chen S., Xu Y.J., Miao Z.H., Ma Y., Qian H.S., Lu Y., Zha Z.B. (2019). Charge reversal induced colloidal hydrogel acts as a multi-stimuli responsive drug delivery platform for synergistic cancer therapy. Mater. Horiz..

[283] Maitz M.F., Sperling C., Wongpinyochit T., Herklotz M., Werner C., Seib F.P. (2017). Biocompatibility assessment of silk nanoparticles: Hemocompatibility and internalization by human blood cells. Nanomedicine.

[284] Omenetto F.G., Kaplan D.L. (2010). New opportunities for an ancient material. Science.

[285] Seib F.P., Pritchard E.M., Kaplan D.L. (2013). Self-assembling doxorubicin silk hydrogels for the focal treatment of primary breast cancer. Adv. Funct. Mater..

[286] Wu P., Liu Q., Wang Q., Qian H., Yu L., Liu B., Li R. (2018). Novel silk fibroin nanoparticles incorporated silk fibroin hydrogel for inhibition of cancer stem cells and tumor growth. Int. J. Nanomed..

[287] Wu H., Liu S., Xiao L., Dong X., Lu Q., Kaplan D.L. (2016). Injectable and pH-Responsive Silk Nanofiber Hydrogels for Sustained Anticancer Drug Delivery. ACS Appl. Mater. Interfaces.

[288] He W., Li P., Zhu Y., Liu M., Huang X., Qi H. (2019). An injectable silk fibroin nanofiber hydrogel hybrid system for tumor upconversion luminescence imaging and photothermal therapy. New J. Chem..

[289] Ribeiro V.P., Silva-Correia J., Goncalves C., Pina S., Radhouani H., Montonen T., Hyttinen J., Roy A., Oliveira A.L., Reis R.L. (2018). Rapidly responsive silk fibroin hydrogels as an artificial matrix for the programmed tumor cells death. PLoS ONE.

[290] Schaal J.L., Li X., Mastria E., Bhattacharyya J., Zalutsky M.R., Chilkoti A., Liu W. (2016). Injectable polypeptide micelles that form radiation crosslinked hydrogels in situ for intratumoral radiotherapy. J. Control. Release.

[291] Poursaid A., Jensen M.M., Nourbakhsh I., Weisenberger M., Hellgeth J.W., Sampath S., Cappello J., Ghandehari H. (2016). Silk-Elastinlike Protein Polymer Liquid Chemoembolic for Localized Release of Doxorubicin and Sorafenib. Mol. Pharm..

[292] Gustafson J.A., Price R.A., Greish K., Cappello J., Ghandehari H. (2010). Silk-elastin-like hydrogel improves the safety of adenovirus-mediated gene-directed enzyme-’prodrug therapy. Mol. Pharm..

[293] Hoffman A.S. (2012). Hydrogels for biomedical applications. Adv. Drug Deliv. Rev..

[294] Naahidi S., Jafari M., Logan M., Wang Y., Yuan Y., Bae H., Dixon B., Chen P. (2017). Biocompatibility of hydrogel-based scaffolds for tissue engineering applications. Biotechnol. Adv..

[295] Steinwachs M., Cavalcanti N., Mauuva Venkatesh Reddy S., Werner C., Tschopp D., Choudur H.N. (2019). Arthroscopic and open treatment of cartilage lesions with BST-CARGEL scaffold and microfracture: A cohort study consecutive patients. Knee.

[296] Elstad N.L., Fowers K.D. (2009). OncoGel (ReGel/paclitaxel) - Clinical applications for a novel paclitaxel delivery system. Adv. Drug Deliv. Rev..

[297] Shalhoub J., Hinchliffe R.J., Powell J.T. (2013). The world of legoo assessed: A short systematic and critical review. Eur. J. Vasc. Endovasc. Surg..

[298] Moreno E., Schwartz J., Larrañeta E., Nguewa P.A., Sanmartín C., Agüeros M., Irache J.M., Espuelas S. (2014). Thermosensitive hydrogels of poly(methyl vinyl ether-co-maleic anhydride) - Pluronic^®^ F127 copolymers for controlled protein release. Int. J. Pharm..

[299] Hwang M.E., Black P.J., Elliston C.D., Wolthuis B.A., Smith D.R., Wu C.C., Wenske S., Deutsch I. (2018). A novel model to correlate hydrogel spacer placement, perirectal space creation, and rectum dosimetry in prostate stereotactic body radiotherapy. Radiat. Oncol..

[300] Rao A.D., Feng Z., Shin E.J., He J., Waters K.M., Coquia S., DeJong R., Rosati L.M., Su L., Li D. (2017). A Novel Absorbable Radiopaque Hydrogel Spacer to Separate the Head of the Pancreas and Duodenum in Radiation Therapy for Pancreatic Cancer. Int. J. Radiat. Oncol. Biol. Phys..

